# Reconstruction of Gene Regulatory Modules in Cancer Cell Cycle by Multi-Source Data Integration

**DOI:** 10.1371/journal.pone.0010268

**Published:** 2010-04-21

**Authors:** Yuji Zhang, Jianhua Xuan, Benildo G. de los Reyes, Robert Clarke, Habtom W. Ressom

**Affiliations:** 1 Lombardi Comprehensive Cancer Center, Georgetown University, Washington, D. C., United States of America; 2 Department of Electrical and Computer Engineering, Virginia Polytechnic Institute and State University, Arlington, Virginia, United States of America; 3 School of Biology and Ecology, University of Maine, Orono, Maine, United States of America; University College Dublin, Ireland

## Abstract

**Background:**

Precise regulation of the cell cycle is crucial to the growth and development of all organisms. Understanding the regulatory mechanism of the cell cycle is crucial to unraveling many complicated diseases, most notably cancer. Multiple sources of biological data are available to study the dynamic interactions among many genes that are related to the cancer cell cycle. Integrating these informative and complementary data sources can help to infer a mutually consistent gene transcriptional regulatory network with strong similarity to the underlying gene regulatory relationships in cancer cells.

**Results and Principal Findings:**

We propose an integrative framework that infers gene regulatory modules from the cell cycle of cancer cells by incorporating multiple sources of biological data, including gene expression profiles, gene ontology, and molecular interaction. Among 846 human genes with putative roles in cell cycle regulation, we identified 46 transcription factors and 39 gene ontology groups. We reconstructed regulatory modules to infer the underlying regulatory relationships. Four regulatory network motifs were identified from the interaction network. The relationship between each transcription factor and predicted target gene groups was examined by training a recurrent neural network whose topology mimics the network motif(s) to which the transcription factor was assigned. Inferred network motifs related to eight well-known cell cycle genes were confirmed by gene set enrichment analysis, binding site enrichment analysis, and comparison with previously published experimental results.

**Conclusions:**

We established a robust method that can accurately infer underlying relationships between a given transcription factor and its downstream target genes by integrating different layers of biological data. Our method could also be beneficial to biologists for predicting the components of regulatory modules in which any candidate gene is involved. Such predictions can then be used to design a more streamlined experimental approach for biological validation. Understanding the dynamics of these modules will shed light on the processes that occur in cancer cells resulting from errors in cell cycle regulation.

## Introduction

Cell division, ageing, and death are intricately regulated processes that depend on the balance between various growth promoting and inhibiting signals. The intricacies of these processes are defined by complex genetic programs that allow certain genes to be expressed in a tightly regulated manner. Errors in regulation cause uncontrolled cell proliferation, a universal property of tumors. This characteristic is driven by genes that exhibit abnormal activities in tumor cells, many of which have important roles in transducing growth-regulating signals to the nucleus and interfacing these signals to modify gene expression. While this signaling inevitably contributes to the proliferative capacity of tumor cells, it is often conceived to do so in a hierarchical manner, by amplifying the activity of afferent signaling, ultimately converging on those genes that control cell cycle progression.

Advances in cancer research during recent years have begun to uncover the intricate genetic programming of cell cycle progression. Expression levels of thousands of genes fluctuate throughout the cancer cell cycle [1], [2]. Periodic transcriptional activities of many genes involved in cell growth, DNA synthesis, spindle pole body duplication, and transit through the cell cycle have each been observed [3]. The transcriptional regulatory networks (TRNs) associated with these activities have been extensively investigated [4], [5], [6], [7], [8]. Further characterization of the genome-wide transcriptional programming of the mammalian cell cycle is a critical step toward understanding the basic cell cycle processes and their precise roles in cancer.

Cell cycle gene expression data obtained from Hela cells have been analyzed with several clustering methods and the genes organized into functional and regulatory groups [1], [2]. Based on these studies, establishing a robust inference regarding the regulatory relationships between a certain transcription factor and its putative target gene(s) could be better accomplished by combining gene expression data with information on transcription factor binding sites and the possible types of interaction based on existing biological knowledge [9]. Transcriptional activation or repression depends on the recognition of specific promoter element sequences by the DNA-binding regulatory protein. How a specific combination of these proteins associates with genes across a genome is referred to as TRN. Therefore, it is important to investigate how these periodic patterns are regulated within the context of TRN of cell cycling in cancer cells.

Reverse engineering of a global TRN remains challenging due to several limitations including (1) the high dimensionality of living cells where tens of thousands of genes act at different temporal and spatial combinations, (2) each gene interacts virtually with multiple partners either directly or indirectly, thus possible relationships are dynamic and non-linear, (3) current high-throughput technologies generate data that involve a substantial amount of noise, and (4) the sample size is extremely low compared with the number of genes [10]. Decomposing a TRN into a small set of recurring regulatory modules (*e.g.*, network motifs) is a promising strategy to address this challenge.

We describe the development of an innovative computational framework that infers complex TRNs by integrating biological data from multiple sources and utilizing the concept of network motif modular analysis. The novelty of this computational framework resides in the decomposition of a complex biological network into dynamically simple but well characterized network motifs, and the ability to integrate disparate biological data to derive these network motifs. The inferred modules provide a rational basis for generating new hypotheses for subsequent experimental validation. We demonstrate the capability of this computational framework to infer regulatory modules associated with the cell cycle progression in Hela cells by combining information from time-course gene expression experiments [2], protein-protein interactions (PPI) [11], [12], [13], [14], [15], [16], [17], [18], [19], [20], [21], [22], protein-DNA interactions (PDI) [23], and gene ontology (GO) [24].

Compared with our previously reported strategy, which was applied to TRN inference in the yeast cell cycle [25], this new scheme includes an integrative use of PPI and PDI data (hereafter called molecular interaction data) from thirteen publically available databases coupled with the detection of significant network motifs for each transcription factor. Implementation of this new scheme significantly expanded the scope of the networks that incorporate deeper sets of known and valuable biological evidence. Moreover, we have introduced a new cluster validity method that utilizes the GO annotation to calculate the similarity of any given pair of genes in a cluster. The partition with the highest similarity score is selected as the optimal cluster. Small TRN modules (*i.e.*, network motifs) are readily interpretable and have the potential to provide insights into new hypotheses. If a gene cluster is involved in the network motif of a transcription factor, and most genes have evidence that they are regulated by that particular transcription factor, it is most likely that other genes in this cluster have similar regulatory relationships with that particular transcription factor. The inference capability of our refined computational framework is verified by various analyses including gene set enrichment analysis (GSEA), binding site enrichment analysis (BSEA), and additional literature survey.

## Results

### Overview of the data integration framework

We considered two different layers of networks in each TRN based on the analysis of Hela cell cycle data. First is the physical network that includes PPIs and PDIs at the factor-gene binding level. Second is the functional network that incorporates the consequences of these physical interactions, such as the activation or repression of transcription. We used three types of data to reconstruct the TRN, namely PPIs derived from a collection of PPI databases, PDIs from the TRANSFAC database, and the time course gene expression profiles as published by [2]. The first two data sources provided direct network information to constrain the TRN model. The gene expression profiles provided an unambiguous measurement on the causal effects of the TRN model. GO annotation describes the similarities between genes within one network, which facilitates further characterization of the relationships between genes. The goal was to discern dependencies between the gene expression patterns and the physical inter-molecular interactions revealed by complementary data sources.

**Figure 1:**
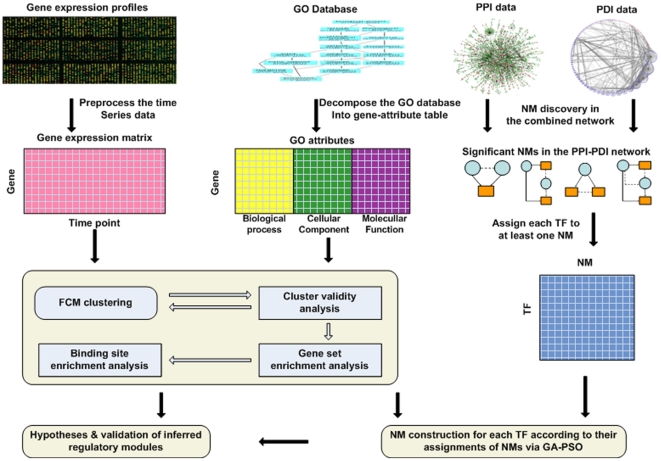
Schematic overview of the computational framework used for the network motif regulatory module inference. Gene expression patterns were first clustered into biologically meaningful groups by FCM; GO category information of genes was used to determine the optimal cluster number. To evaluate the gene clusters, GSEA was performed on the optimal clusters. Additionally, significant network motifs detected in the combined network of PPI and PDI were then assigned to each transcription factor. After the gene clusters are formed and transcription factors were assigned to network motif categories, the connections between transcription factors and gene clusters were inferred by training RNNs that mimic the topology of the network motifs that transcription factors are assigned to. Finally, the inferred network motifs were validated by BSEA and literature results.

The framework model for TRN inference by multi-layer data integration is illustrated in Figure 1. Besides data pre-processing, three successive steps were involved in this framework as outlined in the following:

#### Gene clustering

Genes with similar expression profiles were represented by a cluster to address the scalability problem in TRN inference [26]. The assumption is that a subset of genes that are related in terms of expression (co-regulated) can be grouped together by virtue of a unifying cis-regulatory element(s) associated with a common transcription factor regulating each and every member of the cluster (co-expressed) [27]. GO information was utilized to define the optimal number of clusters with respect to certain broad functional categories. Since each cluster mainly represents one broad biological or process category as evaluated by FuncAssociate [28]), the regulatory network implies that a given transcription factor is likely to be involved in the control of a group of functionally related genes [29].

#### Network motif assignment to transcription factor

To reduce the complexity of the inference problem, network motifs were utilized instead of a global TRN inference. The significant network motifs in the combined molecular interaction network were first established and assigned to at least one transcription factor. These associations were further used to reconstruct the regulatory modules.

#### Construction of network motifs for transcription factor

For each transcription factor assigned to a network motif, a genetic algorithm (GA) generated candidate gene clusters for attribution to a transcription factor based on the relationships established by the network motif. A recurrent neural network (RNN) was trained to model a TRN that mimics the associated network motif. GA generated the candidate gene clusters, and particle swarm optimization (PSO) was used to configure the parameters of the RNN. Parameters were selected to minimize the root mean square error (RMSE) between the output of the RNN and the target gene cluster's expression pattern. The RMSE was returned to GA to produce the next generation of candidate gene clusters. Optimization continued until either a pre-specified maximum number of iterations was completed or a pre-specified minimum RMSE was reached. The procedure was repeated for all transcription factors. Biological knowledge from databases was used to evaluate the predicted results.

### Establishment of optimum number of biologically significant clusters by cluster validity measurement

Genes that belong to similar or related functional categories and that exhibit similar patterns of transcription are likely to be regulated by the same mechanism [30]. Coordinately expressed genes are likely to be unified by common cis-regulatory elements and their cognate transcription factor(s) [31], [32] but this relationship is often easily discernible only in cases where the cluster is comprised of highly to moderately expressed genes. Moreover, in high dimensional data spaces these single correlations are noisy and the underlying correlation structure of the data can be complex [10]. Genes assigned to the same or related functional categories based on gene ontology are also likely to be regulated by a common transcription factor [33]. Integrated analysis of transcript profile data and gene ontology annotation is a more robust approach for network prediction than a uni-dimensional approach based on a single layer of information such as univariate correlation measures.

**Figure 2:**
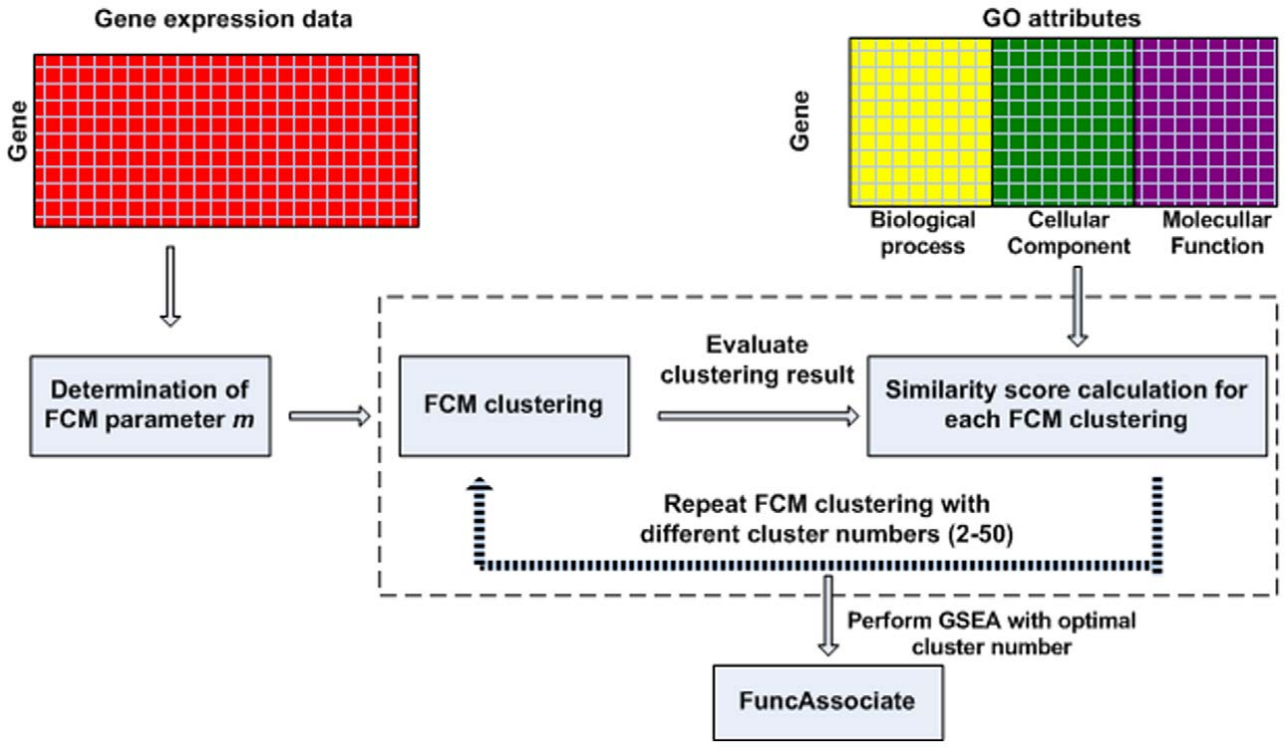
The FCM clustering scheme. The scheme illustrates the process of grouping genes into biologically meaningful clusters. The gene expression data were first utilized to find the optimal m value for FCM clustering. With the optimal m value, FCM clustering was performed on gene expression data for cluster numbers ranging from 2 to 50. The similarity scores of all pairs of genes in each cluster of one partition are averaged and denoted as overall similarity score for one cluster partition. The partition with the highest similarity score was selected as the optimal one. GSEA was performed using FuncAssociate to evaluate the gene clusters formed using the optimal cluster number.

A total of 846 genes associated with the control of cell cycle have been identified previously in Hela cells [2]. We further partitioned these genes into more specific functional groups (Figure 2) by fuzzy c-means clustering (FCM) [34]. In comparison to traditional K-means clustering, this scheme provides a more robust strategy that allows genes with similar expression patterns to be placed in the same cluster with much reduced background noise [26]. FCM clustering involves two empirical parameters: fuzziness parameter *m* and number of clusters *c*. The optimal value of *m* for the dataset used in this study was 1.1548, which was determined based on the method proposed by Dembele and Kastner [35].

The optimal cluster number was determined by the semantic similarity between any gene pair in a single cluster. This is a knowledge-driven method that aims to estimate the optimal cluster partition from a collection of candidate partitions and enhances the predictive reliability and biological relevance of the output. Semantic similarity between gene pairs was calculated by combining the similarity scores between the GO terms assigned to each gene. Relevance similarity measures were used to compute similarity with respect to the assigned GO terminologies [36]. The similarity score of all pairs of genes in each cluster of one partition were averaged and denoted as the overall similarity score for that particular cluster partition.

**Figure 3:**
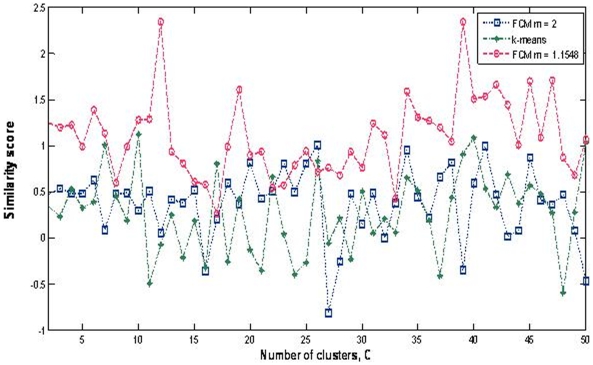
Clustering results obtained using K-mean and FCM algorithms. Three clustering results were plotted: k-means clustering and FCM clustering with two *m* values (*m* is the fuzziness parameter): default value (*m* = 2) and optimal value (*m* = 1.1548).

The cluster validity assessment method considered all three ontology branches (cellular component, molecular function, and biological process) to calculate the similarity scores. The partition with the highest similarity score was selected as the optimal partition (Figure 3). We compared the performance of FCM clustering with the K-mean clustering with respect to two different *m* values. One is a default value of 2 and the other is based on the optimal value of 1.1548 (Figure 2). From this analysis, we observed that FCM clustering with the optimal *m* value gives the best similarity score. The highest similarity score was obtained with 39 clusters, indicating an optimal condition to reduce the search space for TRN inference.

To evaluate the optimal clusters selected based on GO, GSEA was applied using the optimal value (Table S1). Each cluster was enriched in specific biological categories. To further evaluate the biological significance of the established clusters, GO information was used to determine whether the clusters have significant enrichment of one or more terms by using the FuncAssociate program [28]. This strategy made use of a subset of genes as input to produce a ranked list (by P-values) of the GO attributes that are enriched among the input gene subset [24]. The output gave the GO terms that were significantly enriched in each cluster among all genes (equal to the total 26,512 human genes in the FuncAssociate program).

Following this scheme, the total set of genes involved in cell cycle regulation was further subdivided into 39 clusters (Table S1). Of these clusters, 31 were clearly associated with GO categories that imply a more specific function that unifies the members of one but not other clusters, thereby establishing more direct relationships among certain smaller sub-groups of genes. For example, clusters 29 and 8 can both be associated with pre-mitotic, mitotic and post-mitotic events (M-phase). However, members of cluster 8 can be distinguished from the members of cluster 29 by virtue of their specific roles in chromosome doubling (DNA replication) and cytokinesis. Conversely, members of cluster 29 can be distinguished from the members of cluster 8 by virtue of their specific roles in spindle fiber assembly and disassembly.

Biological significance of these highly specific functional relationships, established by our clustering scheme, can further be extended in terms of relationships within the regulatory context. For instance, members of both clusters 29 and 8 have been identified previously as direct downstream targets of E2F factors (Ren et al., 2002). Similar relationships can be established with other clusters such as cluster 32, which is comprised of genes with biochemical roles of a DNA ligase. Thus, the genes in Cluster 32 are involved in processes associated with gap repair or Okazaki fragment processing during DNA replication and chromosome doubling. Previous studies have established that genes associated with this function are under the regulatory control of E2F1 and PCNA (Shibutani et al, 2008; see further details in Table S2).

Based on all these relationships, one specific strength of our current method is its ability to distinguish genes that are related by function in a broad sense and sub-categorizing them into highly specific (narrow) functional categories, resulting in the prediction of regulatory relationships that are consistent with biologically valid relationships.

### Assigning transcription factors to network motifs

TRNs are composed of repeated occurrences of network motifs, which are simple, repeated patterns of conserved biological units ranging from molecular domains to small reaction networks [37]. Each network motif performs a defined information processing function within the network. We focused on three-node network motifs because the majority of the larger size network motifs are composed maximally of three-nodes [38]. The goal was to assign each possible cell cycle control associated transcription factor to at least one network motif according to the combined molecular interaction network. The goal was achieved by building an RNN model for all the possible regulatory genes involved in transcription based on their specific network motif. The RNN output is a model that links each *bona fide* or putative transcriptional regulator with their downstream target genes.

All genes with either direct or indirect roles in the regulation of transcription were first identified from the total set of 846 cell cycle associated genes according to GO categories that denote possible roles in transcription (Ashburner et al., 2000). Candidate genes that remained after filtering other gene function categories are those that were assigned to the following putative functions: transcription factor activity (GO: 0003700), regulation of transcription (GO: 0061019), and transcription factor complex (GO: 0005667). Since GO information alone may not be sufficient to identify the genes with bona fide roles as transcription factors, we further filtered our list of candidate transcription factors by adding another layer of confirmatory information based on the results of PubMed searches. This additional annotation allowed us to validate the GO classification of our candidate genes. The detailed descriptions of GO terms and specific roles in transcription of candidate TFs used in this study in Table S3. Among the 846 cell cycle related genes, 46 were annotated with functions related to transcriptional regulation based on both GO and PubMed databases. These genes were considered as putative transcription factors.

**Figure 4:**
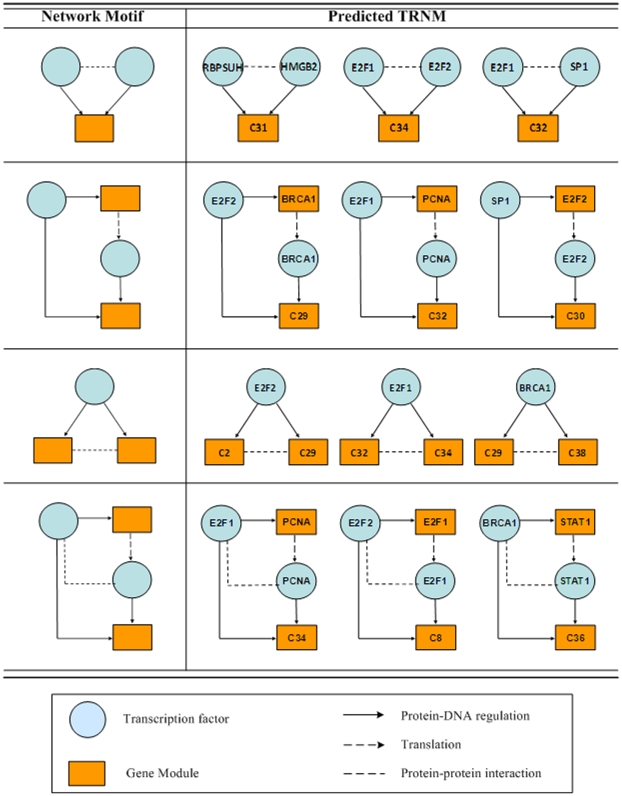
Predicted network motif from known cell cycle dependent genes. The left panel presents the four network motif regulatory modules considered in this study. The right panel depicts inferred transcription factor-target gene relationships for eight cell cycle dependent transcription factors.

In the microarray data, genes are often represented by multiple oligonucleotide probes. Genes represented by probe sets with larger variance were further considered in this study (Zhang et al., 2007). We decomposed the TRN into several network motifs, with each network motif potentially associated with a given transcription factor(s). A total of four network motifs were found to be significant in the combined molecular interaction network (Figure 4), thus each transcription factor was assigned to at least one of these network motifs.

### Inferring network motif regulatory modules between transcription factors and gene clusters

The relationships between transcription factors and gene clusters were determined based on RNN models. For each of the four network motifs (Figure 4), a suitable RNN was built as we previously described [25]. The RNN models were trained using the hybrid genetic algorithm – particle swarm optimization (GA-PSO) to find the downstream gene clusters for all 46 putative transcription factors. Associations between each transcription factor and 39 gene clusters was determined by training the RNN model that mimics the specific network motif for a given transcription factor. Due to a reduction in the computational complexity (mapping between 46 transcription factors and 39 gene clusters instead of 846 genes), the numbers of GA and PSO generations needed to reach the pre-specified minimum RMSE was significantly reduced. The PSO generation for RNN was set to 1000 [39]. The minimum value of RMSE decreased as the number of generations increased (Table 1). The minimum RMSE for GA generations 600 and 800 were 0.077 and 0.075, respectively. Based on 600 GA generations, our inference method successfully assigned all 46 putative transcription factors to their target gene clusters and inferred the most likely transcriptional regulatory network motifs (TRNMs; see Figure 4 for representative TRNMs).

**Table 1:** The experimental results of GA-PSO with RNN.

GA generations	Average RMSE	Minimum RMSE
100	1.27	0.78
200	0.84	0.40
400	0.62	0.12
600	0.35	0.077
800	0.31	0.075

The average and least RMSEs obtained between the output of RNN and the measured expression pattern for the gene clusters are shown as the number of GA generation is varied from 100 to 800.

The validity and accuracy of the network depicted by the TRNMs can be assessed by comparison with a network model constructed based on actual biological data. In the absence of such information, we performed an initial validation of the network by searching for known gene connections in databases. Based on the network motif module prediction results, we collected literature evidence from the NCBI and TRANSFAC [40] databases. We reviewed each predicted network motif and examined the relationships between the transcription factor and its target gene cluster(s). Subsequent analysis was performed under the basic assumption that the inferred network motif is more likely to be biologically meaningful if the transcription factors therein are correlated with the enriched biological functions in the downstream clusters.

Significant network motifs resulting from the survey of available literature cell cycle dependent genes such as *E2F1, E2F2, SP1, BRCA1, STAT1, PCNA, RBPSUH*, and *HMGB2* are listed in Figure 4. Based on the combined information, the biological implication of the network can be explained. For instance, *E2F* is a transcription factor that plays a crucial role in cell-cycle progression in mammalian cells [41]. *E2F1*, which contains two overlapping *E2F*-binding sites in its promoter region, is activated at the G1/S transition in an E2F-dependent manner. *E2F2* interacts with certain elements in the *E2F1* promoter and both genes are involved in DNA replication and repair [42], cytokinesis, and tumor development [43]. According to the GSEA results, Cluster 8 is enriched with genes involved in mitosis and cytokinesis, and Cluster 34 is enriched with genes involved in several functional categories associated with tumor development. As shown in Figure 4, both Cluster 8 and 34 are predicted to be regulated by *E2F1* and *E2F2*, and these results are in agreement with previous reports based on biological data [41], [43].

Our analysis predicts that *E2F1* and *PCNA* are components of the same network. Both of these genes are involved in the regulation of clusters 32 and 34. The best understood molecular function of the *PCNA* protein is its role in the regulation of eukaryotic DNA polymerase delta processivity, which ensures the fidelity of DNA synthesis and repair [44]. However, recent studies have provided evidence that the *PCNA* protein also functions as a direct repressor of the transcriptional coactivator p300 [45]. Another study shows that *PCNA* represses the transcriptional activity of retinoic acid receptors (*RAR*s) [46]. Thus, the involvement of these genes in the same network, as predicted by our network inference algorithm, is strongly supported by knowledge of regulatory relationships already established in experimental data. The results of our prediction are in agreement with these reports since both Clusters 8 and 32 are enriched with genes involved in DNA synthesis and regulatory processes.

We took three approaches to investigate further whether the genes predicted to be regulated by *E2F* genes in Clusters 8, 32 and 34 are validated in classical non-genome wide methods. First, we investigated how many “known” *E2F1* and *E2F2* targets are predicted by our proposed method. According to Bracken *et al*. [47], 130 genes were reviewed as *E2F* targets, 44 of which were originally identified by classical, non-genome-wide approaches. Since we restricted our analysis to the 846 cell cycle related genes, 45 genes matched the *E2F* target genes listed in ref. [47], 21 of which were known from studies using classical molecular biology analyses. The gene targets predicted by our method match 15 of 45 genes, all 15 of which are among those found originally using standard molecular biology experiments. One possible reason is that genome-wide approaches are usually highly noisy and inconsistent across different studies. The detailed information about these genes is listed in Table S4.

Second, we wanted to see whether our predicted gene target clusters are enriched in the corresponding binding sites for the transcription factors in their upstream region. For both *E2F1* and *E2F2*, 7 out of 17 genes in Cluster 8 contain binding sites in their upstream regions as confirmed by data in the SABiosciences database (http://www.sabiosciences.com/chipqpcrsearch.php?app=TFBS).

**Figure 5:**
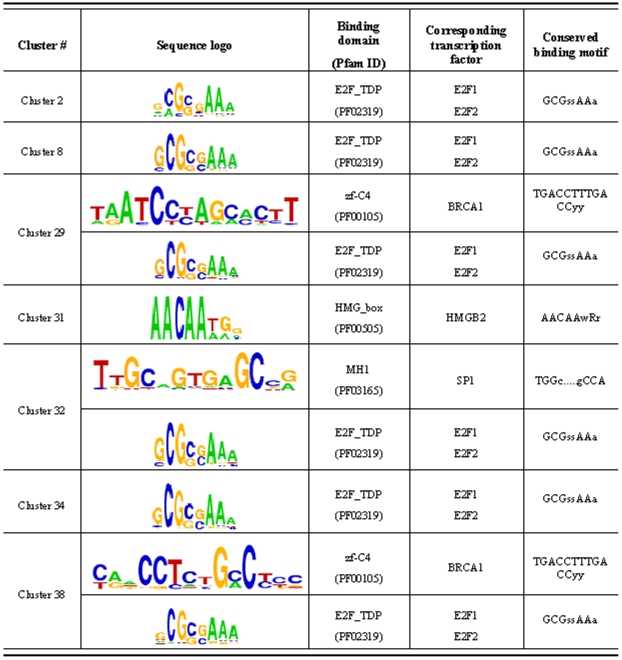
Binding site enrichment analysis for gene clusters. Sequence logos represent the motif significantly overrepresented in individual gene cluster associated with their predicted upstream transcription factors, according to the WebMOTIFS discovery algorithm [48]. Individual base letter height indicates level of conservation within each binding site position. Conserved binding motifs are the conserved binding sequences used in the WebMOTIFS discovery algorithm.

Finally, we determined how many genes in the gene clusters have *E2F* binding sites. We applied the motif discovery tool, WebMOTIFS [48] to find shared motifs in the gene clusters predicted to the *E2F* targets using binding site enrichment analysis (BSEA). The results revealed that a motif called E2F_TDP, GCGSSAAA, is identified as the most significant motif among gene clusters 2, 8, 29, 31, 32 and 34. Unfortunately, for Clusters 30 and 36 the number of genes in these clusters is too small for WebMOTIFS analysis. All these gene clusters are predicted to the downstream targets of *E2F*. For instance, 43 out of 52 genes in Cluster 2 have putative *E2F* binding sites in their upstream regions. The detailed information of BSEA results is shown in Figure 5. For those TRNMs for which two transcription factors are involved, we also find these downstream gene clusters are enriched in both the binding site sequence motifs. For instance, Cluster 32 is enriched in both E2F_TDP and MH1 motifs, corresponding to the two transcription factors in the TRNM: E2F1 and SP1. These BSEA results strongly support our inference results.

**Figure 6:**
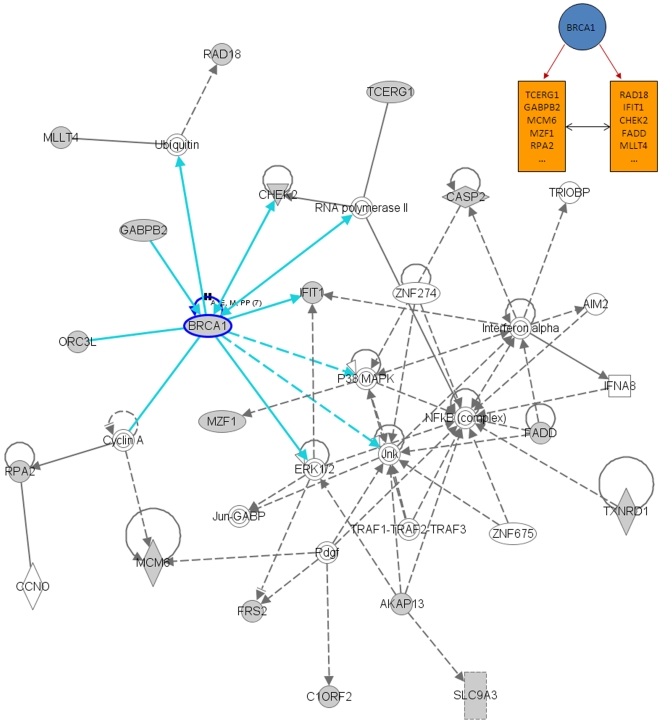
Ingenuity analysis for BRCA1-related network motif: A predicted network motif, where BRCA1 regulates two clusters which interact with each other (top right corner), and a network reconstructed by the IPA software. Shaded genes are genes identified in the network motif and others are those associated with the identified genes based on pathway analysis.

**Figure 7:**
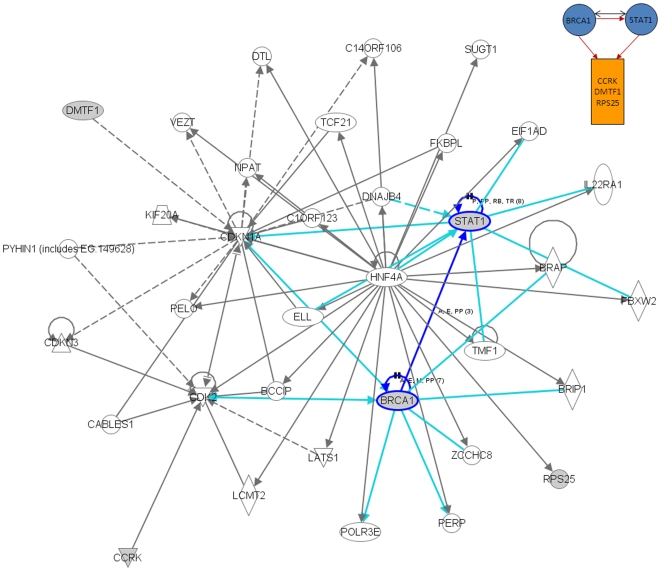
Ingenuity analysis for BRCA1 and STAT1-related network motif: A predicted network motif, in which BRCA1 and STAT1 regulate all three genes in Cluster 36 (top right corner), and a network reconstructed by the IPA software. Shaded genes are genes identified in the network motif and others are those associated with the identified genes based on pathway analysis.

**Figure 8:**
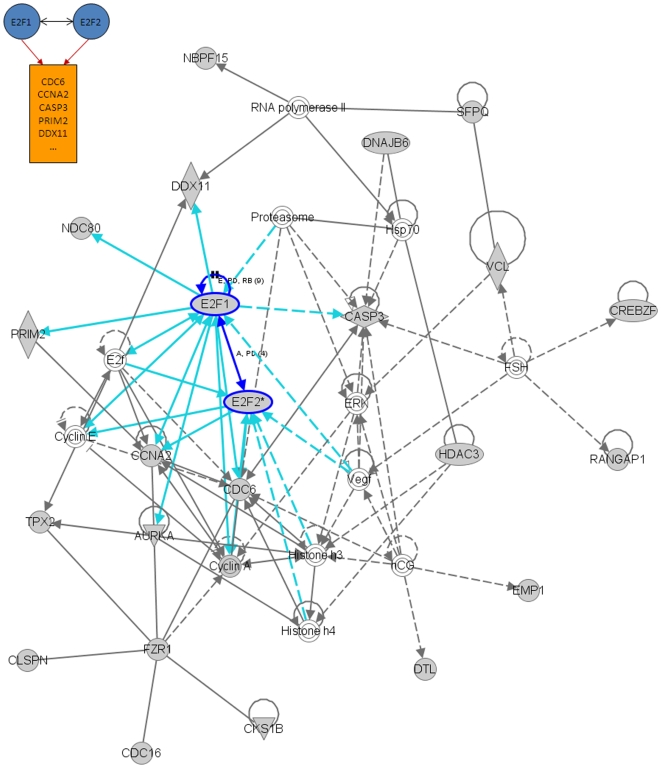
Ingenuity analysis for E2F1 and E2F2-related network motif: A predicted network motif with E2F1 and E2F2 interacting with each other and regulating the genes in Cluster 34 (top left corner), and a network reconstructed by the IPA software. Shaded genes are genes identified in the network motif and others are those associated with the identified genes based on pathway analysis.

**Figure 9:**
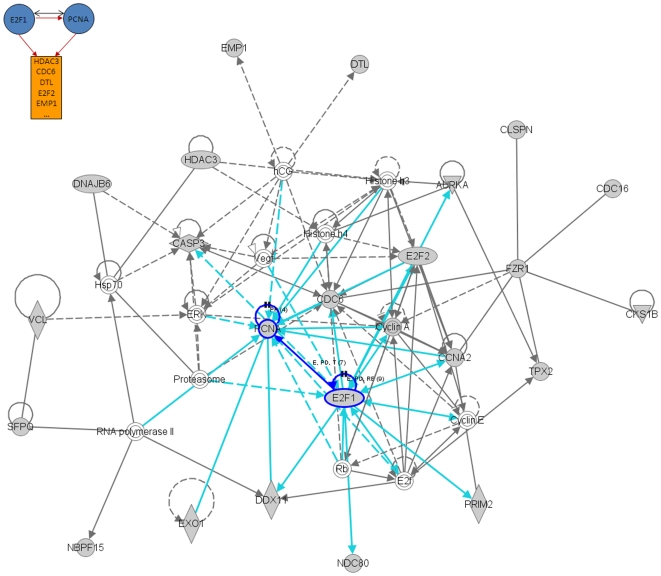
Ingenuity analysis for E2F and PCNA-related network motif: A predicted network motif where E2F2 and PCNA bind together and regulate downstream genes in Cluster 34 (top left corner), and a network reconstructed by the IPA software. Shaded genes are genes identified in the network motif and others are those associated with the identified genes based on pathway analysis.

We also performed an additional analysis of the results presented in Figure 4 using the Ingenuity Pathway Analysis (IPA) software (Ingenuity® Systems, www.ingenuity.com). This tool uses a knowledge base of over one million known functional relationships among proteins. Results of the analysis of the *BRCA1*, *STAT1*, *E2F1*, and *E2F2*-related networks are shown in Figures 6, 7, 8 and 9. These networks were reconstructed based of the putative transcription factors and genes in the predicted network motifs. All the networks confirmed the inferred relationships between TFs and some of the genes in their downstream target clusters. For example, as shown in Figure 6, *BRCA1* regulates two clusters that interact with each other and with the network reconstructed by IPA. Some genes in the clusters show indirect regulations through intermediate genes, such as *BRCA1* acting through *MLLT4* and *RAD18*. Figure 7 depicts a predicted network motif in which *BRCA1* and *STAT1* regulate all three genes in Cluster 36. Figure 8 shows a predicted network motif with *E2F1* and *E2F2* interacting with each other and regulating the genes in Cluster 34. Figure 9 presents a motif where *E2F2* and *PCNA* bind together to activate expression of downstream genes in Cluster 34. For all the other predicted network motifs, the networks reconstructed by the IPA software are presented in the Figures S1, Figure S2, Figure S3, Figure S4, Figure S5, Figure S6, Figure S7 and Figure S8. The notable consistency between IPA and the results from our method indicates that our approach can generate realistic hypotheses for further biological experimental validation.

## Discussion

Reconstruction of TRNs is one of the major challenges in the post-genomics era of biology. In this study, we focused on two broad issues in TRN inference: (1) development of an analysis method that utilizes multiple types of data and (2) network analysis at the network motif level. Based on the information presented, we propose a data integration approach that effectively infers the gene networks underlying certain patterns of gene co-regulation in Hela cell cycling. The predictive strength of this strategy is based on the combined constraints arising from multiple biological data sources, including time course gene expression data, combined molecular interaction network data, and GO category information.

This computational framework allows us to fully exploit the partial constraints that can be inferred from each data source. First, to reduce the inference dimensionalities, the genes were grouped into clusters by FCM, where the optimal fuzziness value was determined by statistical properties of gene expression data. The optimal cluster number was identified by integrating GO category information. Second, the network motif information established from the combined molecular interaction network was used to assign network motif(s) to a given transcription factor. Once the network motif(s) for a transcription factor was identified, a hybrid GA-PSO algorithm was applied to search for target gene clusters that may be regulated by that particular transcription factor. This search was guided by the successful training of a RNN model that mimics the regulatory network motif(s) assigned to the transcription factor. The effectiveness of this method was illustrated via eight well-studied cell cycle dependent transcription factors (Figure 4). The upstream BSEA indicated that the proposed method has the potential to identify the underlying regulatory relationships between transcription factors and their downstream genes at the network motif level. This demonstrates that our approach can serve as a method for analyzing multi-source data at the network motif level.

Compared to the approach developed in [49], our proposed method has several advantages. First, our method performs the inference of TRNs from genome-wide expression data together with other biological knowledge. It has been shown that mRNA expression data alone cannot reflect all the activities in one TRN. Additional information will help constrain the search space of causal relationships between transcription factors and their downstream genes. Second, we decompose the TRN into well characterized functional units - network motifs. Each transcription factor is assigned to specific network motif(s), which is further used to infer the downstream target genes. We not only reduce the search space in the inference process, but also provide experimental biologists the regulatory modules for straightforward validation, instead of one whole TRN containing thousands of genes and connections as is often generated by IPA. Third, we group the genes into functional groups that are potentially regulated by one common transcription factor. The proposed approach reduces the noise in mRNA expression data by incorporating gene functional annotations (*e.g.*, GO).

In summary, we demonstrate that our method can accurately infer the underlying relationships between transcription factor and the downstream target genes by integrating multi-sources of biological data. As the first attempt to integrate many different types of data, we believe that the proposed framework will improve data analysis, particularly as more data sets become available. Our method could also be beneficial to biologists by predicting the components of the TRN in which their candidate gene is involved, followed by designing a more streamlined experiment for biological validation.

## Materials and Methods

### Data sources

The Hela cell cycle data used in the study [2] consists of five time courses (114 total arrays). RNA samples were collected for points (typically every 1–2 h) for 30 h (Thy-Thy1), 44 h (Thy-Thy2), 46 h (Thy-Thy3), 36 h (Thy-Noc), or 14 h (shake) after the synchronous arrest. The cell-cycle related gene set contains 1,134 clones corresponding to 874 UNIGENE clusters (UNIGENE build 143). Of these, 1,072 have corresponding Entrez gene IDs, among which 226 have more than one mapping to clones. In total, 846 genes were used for TRN inference. Also, we choose the Thy-Thy3 time course gene expression pattern for 846 genes, since it has the largest number of time points (47).

Protein-protein interations in human cells are extracted from twelve publicly available large-scale protein interaction maps, seven of which are based on information from scientific literature literature-based, three on orthology information, and two on results of previous yeast two-hybrid (Y2H) analyses. The analysis is restricted to binary interactions in order to make consistent Y2H-based interactions and the remaining maps. Detailed information about the twelve maps is shown in Table 2. To merge twelve interaction maps into one combination map, all proteins are mapped to their corresponding Entrez gene IDs. The human PDI data is extracted from the TRANSFAC database (http://www.gene-regulation.com/pub/databases.html; [23]). The data set consists of 20,473 protein pairs connected by PPIs and 2,546 protein pairs connected as PDIs. The human interaction network related to the 846 genes is extracted based on the interactions among these genes and constructed a network with 1,328 PPIs and 569 PDIs. The analysis is based on network representation of PPIs and PDIs. A node represents both the gene and its protein product. A PPI is represented by a bi-directed edge connecting the interacting proteins. A PDI is an interaction between a transcription factor and its target gene and is represented by a directed edge pointing from the transcription factor to its target gene.

**Table 2:** Networks included in this study.

Networks	Proteins	Interactions	Methods^a^	References	Version^b^
MDC-Y2H	1703	3186	Y2H-ASSAY	Stelzl et al 2005 Cell (Stelzl et al. 2005)	23.09.2005
CCSB-Y2H	1549	2754	Y2H-ASSAY	Rual et al 2005 Nature (Rual et al. 2005)	31.10.2005
HPRD	8788	32776	LITERATURE	Peri et al 2003 Genome Research (Peri et al. 2003)	22.08.2008
DIP	1085	1397	LITERATURE	Salwinski L et al. NAR Database issue 2006 (Salwinski et al. 2004)	01.03.2007
BIND	5286	7394	LITERATURE	Bader et al 2001 NAR (Bader et al. 2001)	01.03.2007
BioGrid	7953	24624	LITERATURE	Stark et al 2006 NAR (Stark et al. 2006)	22.08.2008
IntAct	7273	19404	LITERATURE	Hermjakob et al 2004 NAR (Hermjakob et al. 2004)	22.08.2008
COCIT	3737	6580	TEXT-MINING	Ramani et al. 2004 Genome Biology (Ramani et al. 2005)	18.11.2005
REACTOME	1554	37332	LITERATURE	Joshi-Tope,G et al. 2005 NAR (Joshi-Tope et al. 2005)	01.03.2007
ORTHO	6225	71466	ORTHOLOGY	Lehner et al 2003 Genome Biology (Lehner and Fraser 2004)	17.11.2005
HOMOMINT	4127	10174	ORTHOLOGY	Persico et al 2005 BMC Bioinformatics (Persico et al. 2005)	01.06.2006
OPHID	4785	24991	ORTHOLOGY	Brown et al 2005 Bioinformatics (Brown and Jurisica 2005)	14.12.2005

The table displays the number of proteins and the number of interactions derived from each map.

a Methods refers to the approach taken from the construction of the corresponding map.

b Version describes the date of data downloaded for each dataset.

The GO term definitions are taken from the monthly release from August 2008.

### Data preprocessing

From the time course gene expression data, 846 genes were previously identified as cell cycle regulated based on analysis combining a Fourier algorithm and a correlation algorithm [50]. These genes are functionally annotated based on GO information. Missing values in the data are imputed using K-nearest neighbour (KNN) imputation [32]. The expression pattern of each gene is standardized between −1 and 1. Known network motifs are extracted from the combined molecular interaction network.

### Soft clustering method

A soft clustering approach using FCM [35] was used to cluster genes into biologically meaningful groups. The FCM Matlab toolbox [35] was used. Parameters for FCM were set as default except the following two: the fuzziness parameter *m*, and the cluster number *c*.

An empirical method [35] was used to determine *m*; the method determines an adequate value for *m* based on the distribution of distances between genes.

The optimal cluster number *c* was evaluated by the shared GO annotation within one cluster. Semantic similarity between gene products was calculated by combining the similarity scores between the GO terms annotated to each gene product. To estimate GO-based similarity scores of gene products, Schliker's measure was applied to compute GO term similarity. These measures take relevance information into account by combining Lin's and Resnik's similarity measures [51], [52]. The mgeneSim function of the SemSim Package of Bioconductor [53] was used to perform this function. This algorithm calculates pairwise similarity scores for a list of genes with GO annotation available. The larger the similarity score, the more shared functions these genes share.

### Identification of network motifs

All connected subnetworks containing three nodes in the interaction network were collated into isomorphic patterns, and the number of times each pattern occurred was counted. If the number of occurrences is at least five and significantly higher than in randomized networks, the pattern is considered as a network motif. The statistical significance test was performed by generating 1000 randomized networks and computing the fraction of randomized networks in which the pattern appeared at least as often as in the interaction network, as described in detail in [38]. A pattern with p≤0.05 was considered statistically significant. This network motif discovery procedure is performed using the FANMOD software [54].

### Network motif construction for each transcription factor

A RNN was used to construct a model of the network motif for each transcription factor. Due to its capability to capture the nonlinear properties and dynamic relationships, RNNs have been applied for TRN inference [39], [55], [56]. For each of the four significant network motifs in Figure 4, a suitable RNN is built. A detailed description about RNN training can be found in [25].

## Supporting Information

Table S1 39 clusters and their corresponding enriched GO categories.
(0.03 MB PDF)

Table S2 Details of gene clusters considered in this study.
(0.09 MB PDF)

Table S3 A list of 46 transcription factors in human cell cycle selected as candidates to regulate downstream target genes.
(0.03 MB PDF)

Table S4 Identified E2F target genes.
(0.04 MB PDF)

Figure S1 Ingenuity analysis result for a predicted network motif.
(0.24 MB TIF)

Figure S2 Ingenuity analysis result for a predicted network motif.
(0.22 MB TIF)

Figure S3 Ingenuity analysis result for a predicted network motif.
(0.23 MB TIF)

Figure S4 Ingenuity analysis result for a predicted network motif.
(0.25 MB TIF)

Figure S5 Ingenuity analysis result for a predicted network motif.
(0.19 MB TIF)

Figure S6 Ingenuity analysis result for a predicted network motif.
(0.22 MB TIF)

Figure S7 Ingenuity analysis result for a predicted network motif.
(0.19 MB TIF)

Figure S8 Ingenuity analysis result for a predicted network motif.
(0.22 MB TIF)
