# Supplementary material for: Reconstruction of Gene Regulatory Modules in Cancer Cell Cycle by Multi-Source Data Integration
Source: PLoS One. 2010 Apr 21;5(4):e10268. doi: 10.1371/journal.pone.0010268 (PMC2858157; doi:10.1371/journal.pone.0010268)
Supplement: Table S1 — 39 clusters and their corresponding enriched GO categories. (0.03 MB PDF) [file pone.0010268.s001.pdf]

Cluster #	Number of genes in this cluster	Number of genes in this GO category	P-value	GO category	Enriched functional category
1	8	323	1.60E-08	GO:0000278	mitotic cell cycle
	3	5	2.70E-08	GO:0004756	selenide, water dikinase activity
	3	5	2.70E-08	GO:0016781	phosphotransferase activity, paired acceptors
	10	810	1.50E-07	GO:0007049	cell cycle
	8	452	2.20E-07	GO:0022402	cell cycle process
	7	359	7.10E-07	GO:0022403	cell cycle phase
	3	20	3.00E-06	GO:0004571	mannosyl-oligosaccharide 1,2-alpha-mannosidase activity
	3	22	4.10E-06	GO:0015924	mannosyl-oligosaccharide mannosidase activity
	3	29	9.70E-06	GO:0015923	mannosidase activity
	12	2263	5.00E-05	GO:0005524	ATP binding
2	8	323	5.20E-08	GO:0000278	mitotic cell cycle
	8	359	1.20E-07	GO:0022403	cell cycle phase
	13	1436	3.40E-07	GO:0006793	phosphorus metabolic process
	13	1436	3.40E-07	GO:0006796	phosphate metabolic process
	10	810	6.30E-07	GO:0007049	cell cycle
	8	452	6.70E-07	GO:0022402	cell cycle process
	6	292	8.20E-06	GO:0000279	M phase
	10	1187	1.90E-05	GO:0016310	phosphorylation
	31	10329	2.70E-05	GO:0044237	cellular metabolic process
	15	2823	2.70E-05	GO:0032553	ribonucleotide binding
	15	2823	2.70E-05	GO:0032555	purine ribonucleotide binding
	5	218	2.90E-05	GO:0007067	mitosis
	24	6657	3.00E-05	GO:0043283	biopolymer metabolic process
	5	223	3.30E-05	GO:0000087	M phase of mitotic cell cycle
	15	2934	4.30E-05	GO:0017076	purine nucleotide binding
	16	3353	5.10E-05	GO:0000166	nucleotide binding

Cluster #	Number of genes in this cluster	Number of genes in this GO category	P-value	GO category	Enriched functional category
4	3	5	1.00E-09	GO:0006597	spermine biosynthetic process
	3	5	1.00E-09	GO:0008215	spermine metabolic process
	3	6	2.00E-09	GO:0004014	adenosylmethionine decarboxylase activity
	3	8	5.60E-09	GO:0008295	spermidine biosynthetic process
	3	9	8.40E-09	GO:0008216	spermidine metabolic process
	3	12	2.20E-08	GO:0006596	polyamine biosynthetic process
	3	15	4.50E-08	GO:0006595	polyamine metabolic process
	3	32	4.90E-07	GO:0042401	biogenic amine biosynthetic process
	3	36	7.10E-07	GO:0042398	amino acid derivative biosynthetic process
	3	51	2.10E-06	GO:0016831	carboxy-lyase activity
	3	69	5.10E-06	GO:0016830	carbon-carbon lyase activity
	3	71	5.60E-06	GO:0006576	biogenic amine metabolic process
	3	86	1.00E-05	GO:0006575	amino acid derivative metabolic process
	3	94	1.30E-05	GO:0009309	amine biosynthetic process
	3	122	2.90E-05	GO:0044271	nitrogen compound biosynthetic process
5	2	103	0.0002	GO:0009314	response to radiation
6	4	31	2.50E-08	GO:0004707	MAP kinase activity
	5	157	4.70E-07	GO:0031497	chromatin assembly
	4	70	7.10E-07	GO:0004702	receptor signaling protein serine/threonine kinase activity
	5	186	1.10E-06	GO:0006323	DNA packaging
	5	209	1.90E-06	GO:0006333	chromatin assembly or disassembly
	4	100	3.00E-06	GO:0000786	nucleosome
	5	250	4.60E-06	GO:0000785	chromatin
	6	470	6.00E-06	GO:0051276	chromosome organization and biogenesis
	4	139	1.10E-05	GO:0006334	nucleosome assembly
	20	8814	1.70E-05	GO:0043170	macromolecule metabolic process
	4	168	2.30E-05	GO:0005057	receptor signaling protein activity
	5	386	3.70E-05	GO:0044427	chromosomal part
	5	387	3.80E-05	GO:0006325	establishment and/or maintenance of chromatin architecture
	10	2263	4.00E-05	GO:0005524	ATP binding
	27	17624	4.30E-05	GO:0005488	binding
	10	2286	4.40E-05	GO:0032559	adenyl ribonucleotide binding
	10	2392	6.40E-05	GO:0030554	adenyl nucleotide binding
7	2	38	1.80E-05	GO:0019722	calcium-mediated signaling

Cluster #	Number of genes in this cluster	Number of genes in this GO category	P-value	GO category	Enriched functional category
8	3	32	9.20E-07	GO:0000910	cytokinesis
	3	36	1.30E-06	GO:0006270	DNA replication initiation
	4	223	8.70E-06	GO:0000087	M phase of mitotic cell cycle
	3	73	1.10E-05	GO:0008094	DNA-dependent ATPase activity
	3	136	7.30E-05	GO:0006261	DNA-dependent DNA replication
9	9	292	7.60E-09	GO:0000279	M phase
	8	223	1.70E-08	GO:0000087	M phase of mitotic cell cycle
	10	452	2.40E-08	GO:0022402	cell cycle process
	9	359	4.50E-08	GO:0022403	cell cycle phase
	12	810	6.90E-08	GO:0007049	cell cycle
	11	679	1.10E-07	GO:0006259	DNA metabolic process
	8	323	3.00E-07	GO:0000278	mitotic cell cycle
	7	218	3.00E-07	GO:0007067	mitosis
	6	141	4.40E-07	GO:0007218	neuropeptide signaling pathway
	8	470	4.80E-06	GO:0051276	chromosome organization and biogenesis
	26	5551	9.70E-06	GO:0005634	nucleus
	4	73	1.50E-05	GO:0008094	DNA-dependent ATPase activity
	49	16682	1.50E-05	GO:0009987	cellular process
	4	75	1.70E-05	GO:0000775	chromosome, pericentric region
	3	36	5.50E-05	GO:0006270	DNA replication initiation
12	4	14	1.40E-09	GO:0008191	metalloendopeptidase inhibitor activity
	4	60	6.60E-07	GO:0007059	chromosome segregation
	3	20	1.50E-06	GO:0004571	mannosyl-oligosaccharide 1,2-alpha-mannosidase activity
	3	22	2.10E-06	GO:0015924	mannosyl-oligosaccharide mannosidase activity
	3	29	4.90E-06	GO:0000070	mitotic sister chromatid segregation
	3	29	4.90E-06	GO:0015923	mannosidase activity
	3	30	5.40E-06	GO:0000819	sister chromatid segregation

Cluster #	Number of genes in this cluster	Number of genes in this GO category	P-value	GO category	Enriched functional category
15	7	333	5.20E-08	GO:0006260	DNA replication
	7	679	6.10E-06	GO:0006259	DNA metabolic process
	3	36	7.80E-06	GO:0006270	DNA replication initiation
	17	5551	9.40E-06	GO:0005634	nucleus
	4	132	1.20E-05	GO:0005813	centrosome
	4	136	1.30E-05	GO:0006261	DNA-dependent DNA replication
	4	146	1.80E-05	GO:0005815	microtubule organizing center
	7	810	1.90E-05	GO:0007049	cell cycle
	5	323	2.20E-05	GO:0000278	mitotic cell cycle
	2	7	2.30E-05	GO:0045931	positive regulation of mitotic cell cycle
	11	2607	4.70E-05	GO:0043234	protein complex
16	10	1312	1.30E-06	GO:0003700	transcription factor activity
	2	3	3.80E-06	GO:0004146	dihydrofolate reductase activity
	2	4	7.60E-06	GO:0006545	glycine biosynthetic process
	25	11558	2.50E-05	GO:0008152	metabolic process
	10	1870	2.90E-05	GO:0030528	transcription regulator activity
	17	5551	2.90E-05	GO:0005634	nucleus
	2	10	5.70E-05	GO:0009070	serine family amino acid biosynthetic process
17	5	76	1.80E-08	GO:0005643	nuclear pore
	5	85	3.10E-08	GO:0046930	pore complex
	6	188	4.40E-08	GO:0005635	nuclear envelope
	5	104	8.60E-08	GO:0044453	nuclear membrane part
	5	144	4.40E-07	GO:0031965	nuclear membrane
	9	1090	1.40E-06	GO:0044428	nuclear part
	12	2607	7.40E-06	GO:0043234	protein complex
	5	260	7.90E-06	GO:0019932	second-messenger-mediated signaling
	2	5	1.10E-05	GO:0046934	phosphatidylinositol-4,5-bisphosphate 3-kinase activity
	6	598	3.60E-05	GO:0031967	organelle envelope
	6	600	3.70E-05	GO:0031975	envelope
	16	5551	4.80E-05	GO:0005634	nucleus
	12	3215	6.20E-05	GO:0032991	macromolecular complex
	6	679	7.30E-05	GO:0006259	DNA metabolic process

Cluster #	Number of genes in this cluster	Number of genes in this GO category	P-value	GO category	Enriched functional category
18	10	2263	7.90E-05	GO:0005524	ATP binding
19	2	6	4.60E-06	GO:0005521	lamin binding
	2	33	0.00016	GO:0000159	protein phosphatase type 2A complex
20	12	1388	1.30E-06	GO:0006996	organelle organization and biogenesis
	7	386	2.60E-06	GO:0044427	chromosomal part
	5	153	4.70E-06	GO:0007018	microtubule-based movement
	7	454	7.60E-06	GO:0005694	chromosome
	5	174	8.80E-06	GO:0030705	cytoskeleton-dependent intracellular transport
	4	100	2.10E-05	GO:0000786	nucleosome
	15	2871	2.50E-05	GO:0016043	cellular component organization and biogenesis
	2	5	2.50E-05	GO:0004963	follicle-stimulating hormone receptor activity
	4	121	4.30E-05	GO:0003777	microtubule motor activity
21	14	810	1.70E-09	GO:0007049	cell cycle
	29	5551	1.10E-06	GO:0005634	nucleus
	7	298	3.90E-06	GO:0051726	regulation of cell cycle
	7	359	1.30E-05	GO:0022403	cell cycle phase
	2	3	1.40E-05	GO:0004146	dihydrofolate reductase activity
	34	8221	1.60E-05	GO:0043231	intracellular membrane-bounded organelle
	34	8223	1.60E-05	GO:0043227	membrane-bounded organelle
	2	4	2.80E-05	GO:0006545	glycine biosynthetic process

Cluster #	Number of genes in this cluster	Number of genes in this GO category	P-value	GO category	Enriched functional category
22	6	15	8.20E-13	GO:0004957	prostaglandin E receptor activity
	6	19	4.40E-12	GO:0004955	prostaglandin receptor activity
	6	23	1.60E-11	GO:0004953	icosanoid receptor activity
	6	23	1.60E-11	GO:0004954	prostanoid receptor activity
	13	679	8.50E-09	GO:0006259	DNA metabolic process
	9	333	1.20E-07	GO:0006260	DNA replication
	9	359	2.20E-07	GO:0022403	cell cycle phase
	6	136	1.00E-06	GO:0006261	DNA-dependent DNA replication
	9	452	1.50E-06	GO:0022402	cell cycle process
	8	354	2.30E-06	GO:0006281	DNA repair
	11	810	3.90E-06	GO:0007049	cell cycle
	3	13	3.90E-06	GO:0006268	DNA unwinding during replication
	3	15	6.10E-06	GO:0032392	DNA geometric change
	3	15	6.10E-06	GO:0032508	DNA duplex unwinding
	7	292	7.10E-06	GO:0000279	M phase
	8	414	7.40E-06	GO:0006974	response to DNA damage stimulus
	7	323	1.40E-05	GO:0000278	mitotic cell cycle
	3	20	1.50E-05	GO:0004571	mannosyl-oligosaccharide 1,2-alpha-mannosidase activity
	2	3	1.70E-05	GO:0006271	DNA strand elongation during DNA replication
	2	3	1.70E-05	GO:0022616	DNA strand elongation
	3	22	2.10E-05	GO:0015924	mannosyl-oligosaccharide mannosidase activity
	32	6657	2.40E-05	GO:0043283	biopolymer metabolic process
	6	245	3.00E-05	GO:0051301	cell division
	4	73	3.10E-05	GO:0008094	DNA-dependent ATPase activity
	8	505	3.10E-05	GO:0009719	response to endogenous stimulus
	3	29	4.80E-05	GO:0015923	mannosidase activity
	36	8319	5.40E-05	GO:0005515	protein binding
	2	5	5.80E-05	GO:0004963	follicle-stimulating hormone receptor activity

Cluster #	Number of genes in this cluster	Number of genes in this GO category	P-value	GO category	Enriched functional category
24	27	5551	7.00E-07	GO:0005634	nucleus
	17	2346	1.10E-06	GO:0043228	non-membrane-bounded organelle
	17	2346	1.10E-06	GO:0043232	intracellular non-membrane-bounded organelle
	7	333	3.50E-06	GO:0006260	DNA replication
	21	3862	4.40E-06	GO:0044446	intracellular organelle part
	21	3872	4.60E-06	GO:0044422	organelle part
	32	8221	4.90E-06	GO:0043231	intracellular membrane-bounded organelle
	32	8223	4.90E-06	GO:0043227	membrane-bounded organelle
	35	9656	5.50E-06	GO:0043229	intracellular organelle
	35	9661	5.60E-06	GO:0043226	organelle
	9	679	5.60E-06	GO:0006259	DNA metabolic process
	32	8319	6.40E-06	GO:0005515	protein binding
	7	386	9.20E-06	GO:0044427	chromosomal part
	3	28	2.20E-05	GO:0006275	regulation of DNA replication
	7	454	2.60E-05	GO:0005694	chromosome
	2	5	3.70E-05	GO:0005652	nuclear lamina
	10	1084	3.70E-05	GO:0005198	structural molecule activity
	3	34	3.90E-05	GO:0006284	base-excision repair
25	2	3	1.80E-06	GO:0031616	spindle pole centrosome
	4	162	7.30E-06	GO:0005179	hormone activity

Cluster #	Number of genes in this cluster	Number of genes in this GO category	P-value	GO category	Enriched functional category
26	3	3	2.70E-09	GO:0004799	thymidylate synthase activity
	3	3	2.70E-09	GO:0006231	dTMP biosynthetic process
	3	3	2.70E-09	GO:0009157	deoxyribonucleoside monophosphate biosynthetic process
	3	3	2.70E-09	GO:0009162	deoxyribonucleoside monophosphate metabolic process
	3	3	2.70E-09	GO:0009176	pyrimidine deoxyribonucleoside monophosphate metabolic process
	3	3	2.70E-09	GO:0009177	pyrimidine deoxyribonucleoside monophosphate biosynthetic process
	3	3	2.70E-09	GO:0042083	5,10-methylenetetrahydrofolate-dependent methyltransferase activity
	3	3	2.70E-09	GO:0046073	dTMP metabolic process
	3	5	2.70E-08	GO:0006597	spermine biosynthetic process
	3	5	2.70E-08	GO:0008215	spermine metabolic process
	3	5	2.70E-08	GO:0009129	pyrimidine nucleoside monophosphate metabolic process
	3	5	2.70E-08	GO:0009130	pyrimidine nucleoside monophosphate biosynthetic process
	3	5	2.70E-08	GO:0009221	pyrimidine deoxyribonucleotide biosynthetic process
	3	6	5.40E-08	GO:0004014	adenosylmethionine decarboxylase activity
	3	6	5.40E-08	GO:0009263	deoxyribonucleotide biosynthetic process
	3	8	1.50E-07	GO:0008295	spermidine biosynthetic process
	3	9	2.30E-07	GO:0008216	spermidine metabolic process
	3	12	5.90E-07	GO:0006596	polyamine biosynthetic process
	3	14	9.80E-07	GO:0009219	pyrimidine deoxyribonucleotide metabolic process
	3	15	1.20E-06	GO:0006595	polyamine metabolic process
	3	19	2.60E-06	GO:0009262	deoxyribonucleotide metabolic process
	3	32	1.30E-05	GO:0042401	biogenic amine biosynthetic process
	3	33	1.40E-05	GO:0009123	nucleoside monophosphate metabolic process
	3	33	1.40E-05	GO:0009124	nucleoside monophosphate biosynthetic process
	3	36	1.90E-05	GO:0042398	amino acid derivative biosynthetic process
	3	38	2.20E-05	GO:0006221	pyrimidine nucleotide biosynthetic process
	3	51	5.40E-05	GO:0006220	pyrimidine nucleotide metabolic process
	3	51	5.40E-05	GO:0016831	carboxy-lyase activity

Cluster #	Number of genes in this cluster	Number of genes in this GO category	P-value	GO category	Enriched functional category
27	3	9	5.30E-08	GO:0016538	cyclin-dependent protein kinase regulator activity
	9	1388	1.80E-06	GO:0006996	organelle organization and biogenesis
	2	3	2.30E-06	GO:0004146	dihydrofolate reductase activity
	2	4	4.60E-06	GO:0006545	glycine biosynthetic process
	5	386	2.10E-05	GO:0044427	chromosomal part
	2	10	3.40E-05	GO:0009070	serine family amino acid biosynthetic process
	5	454	4.50E-05	GO:0005694	chromosome
	5	470	5.30E-05	GO:0051276	chromosome organization and biogenesis
	6	810	6.40E-05	GO:0007049	cell cycle
28	4	245	3.90E-06	GO:0051301	cell division
29	6	292	8.00E-08	GO:0000279	M phase
	6	323	1.50E-07	GO:0000278	mitotic cell cycle
	4	75	3.40E-07	GO:0005819	spindle
	5	223	7.30E-07	GO:0000087	M phase of mitotic cell cycle
	3	24	8.50E-07	GO:0000922	spindle pole
	2	3	1.80E-06	GO:0031616	spindle pole centrosome
	4	245	3.70E-05	GO:0051301	cell division
30	2	44	8.70E-05	GO:0030693	caspase activity
31	8	638	1.90E-06	GO:0007010	cytoskeleton organization and biogenesis
	4	75	3.30E-06	GO:0000775	chromosome, pericentric region
	10	1388	1.20E-05	GO:0006996	organelle organization and biogenesis
	6	386	1.30E-05	GO:0044427	chromosomal part
	5	238	1.70E-05	GO:0030036	actin cytoskeleton organization and biogenesis
	5	256	2.40E-05	GO:0030029	actin filament-based process
	6	452	3.10E-05	GO:0022402	cell cycle process
	6	454	3.20E-05	GO:0005694	chromosome
	12	2346	4.10E-05	GO:0043228	non-membrane-bounded organelle
	12	2346	4.10E-05	GO:0043232	intracellular non-membrane-bounded organelle
	5	292	4.60E-05	GO:0000279	M phase

Cluster #	Number of genes in this cluster	Number of genes in this GO category	P-value	GO category	Enriched functional category
32	3	9	1.30E-06	GO:0016538	cyclin-dependent protein kinase regulator activity
	6	202	1.20E-05	GO:0019787	small conjugating protein ligase activity
	6	239	3.10E-05	GO:0016881	acid-amino acid ligase activity
	7	386	5.20E-05	GO:0044427	chromosomal part
33	7	679	2.60E-08	GO:0006259	DNA metabolic process
	5	810	4.80E-05	GO:0007049	cell cycle
	4	414	5.70E-05	GO:0006974	response to DNA damage stimulus
	11	6657	7.10E-05	GO:0043283	biopolymer metabolic process
	10	5551	0.00011	GO:0005634	nucleus

Cluster #	Number of genes in this cluster	Number of genes in this GO category	P-value	GO category	Enriched functional category
34	11	292	1.70E-10	GO:0000279	M phase
	10	218	1.80E-10	GO:0007067	mitosis
	10	223	2.20E-10	GO:0000087	M phase of mitotic cell cycle
	10	245	5.60E-10	GO:0051301	cell division
	11	359	1.50E-09	GO:0022403	cell cycle phase
	10	323	8.00E-09	GO:0000278	mitotic cell cycle
	11	452	1.60E-08	GO:0022402	cell cycle process
	3	5	1.50E-07	GO:0006597	spermine biosynthetic process
	3	5	1.50E-07	GO:0008215	spermine metabolic process
	3	6	3.00E-07	GO:0004014	adenosylmethionine decarboxylase activity
	3	8	8.40E-07	GO:0008295	spermidine biosynthetic process
	32	5551	8.90E-07	GO:0005634	nucleus
	3	9	1.30E-06	GO:0008216	spermidine metabolic process
	3	9	1.30E-06	GO:0016538	cyclin-dependent protein kinase regulator activity
	15	1388	1.40E-06	GO:0006996	organelle organization and biogenesis
	9	470	2.60E-06	GO:0051276	chromosome organization and biogenesis
	3	12	3.30E-06	GO:0006596	polyamine biosynthetic process
	43	9656	4.00E-06	GO:0043229	intracellular organelle
	5	100	5.30E-06	GO:0000786	nucleosome
	3	15	6.70E-06	GO:0006595	polyamine metabolic process
	18	2346	1.30E-05	GO:0043228	non-membrane-bounded organelle
	18	2346	1.30E-05	GO:0043232	intracellular non-membrane-bounded organelle
	8	454	1.80E-05	GO:0005694	chromosome
	47	11718	1.80E-05	GO:0044424	intracellular part
	6	225	2.20E-05	GO:0065004	protein-DNA complex assembly
	5	139	2.60E-05	GO:0006334	nucleosome assembly
	4	71	3.10E-05	GO:0006576	biogenic amine metabolic process
	37	8221	3.40E-05	GO:0043231	intracellular membrane-bounded organelle
	23	3862	4.00E-05	GO:0044446	intracellular organelle part
	23	3872	4.10E-05	GO:0044422	organelle part
	5	157	4.70E-05	GO:0031497	chromatin assembly
	9	679	4.90E-05	GO:0006259	DNA metabolic process

Cluster #	Number of genes in this cluster	Number of genes in this GO category	P-value	GO category	Enriched functional category
35	4	19	4.00E-08	GO:0007051	spindle organization and biogenesis
	8	292	6.50E-08	GO:0000279	M phase
	8	323	1.40E-07	GO:0000278	mitotic cell cycle
	7	218	1.60E-07	GO:0007067	mitosis
	7	223	1.90E-07	GO:0000087	M phase of mitotic cell cycle
	8	359	3.20E-07	GO:0022403	cell cycle phase
	7	283	9.20E-07	GO:0007017	microtubule-based process
	8	452	1.80E-06	GO:0022402	cell cycle process
	10	810	2.10E-06	GO:0007049	cell cycle
	5	114	2.30E-06	GO:0000226	microtubule cytoskeleton organization and biogenesis
	6	245	6.20E-06	GO:0051301	cell division
	3	22	9.10E-06	GO:0030261	chromosome condensation
	2	3	1.00E-05	GO:0045136	development of secondary sexual characteristics
	3	32	2.90E-05	GO:0045787	positive regulation of cell cycle
	5	202	3.70E-05	GO:0019787	small conjugating protein ligase activity
36	3	608	9.90E-05	GO:0004674	protein serine/threonine kinase activity
38	5	141	3.20E-06	GO:0007218	neuropeptide signaling pathway
	2	7	5.30E-05	GO:0005123	death receptor binding
