# Supplementary material for: Reconstruction of Gene Regulatory Modules in Cancer Cell Cycle by Multi-Source Data Integration
Source: PLoS One. 2010 Apr 21;5(4):e10268. doi: 10.1371/journal.pone.0010268 (PMC2858157; doi:10.1371/journal.pone.0010268)
Supplement: Table S2 — Details of gene clusters considered in this study. (0.09 MB PDF) [file pone.0010268.s002.pdf]

Gene Symbol	Cluster #	Gene Symbol	Cluster #	Gene Symbol	Cluster #	Gene Symbol	Cluster #
RNPC3	0	HSD17B11	12	ZNF414	21	AOC3	31
BIRC2	1	KLF6	12	ZNFX1	21	APEX2	31
BLM	1	MAN1A2	12	ZRANB2	21	ARHGDIB	31
C14ORF106	1	MID1	12	ZWINT	21	BTBD3	31
C20ORF117	1	MRPL19	12	ANKRD10	22	C14ORF130	31
CDCA7L	1	NCAPH	12	ARHGAP11A	22	CALM3	31
CDCA8	1	NNMT	12	ARHGAP19	22	CENPE	31
CDKN3	1	NT5DC1	12	ARL6IP2	22	CNN2	31
CENPA	1	NUSAP1	12	ATF7IP	22	CTSD	31
CNIH4	1	PDGFA	12	BRD8	22	ERN2	31
COL7A1	1	RAD21	12	BUB1	22	FOXM1	31
CTCFL	1	RCBTB2	12	BUB3	22	HCP5	31
ARGLU1	1	SEPN1	12	CCDC88A	22	HELLS	31
ERN2	1	SFPQ	12	CDC7	22	HMGB3	31
HELLS	1	SLC17A2	12	CDCA5	22	IFIT2	31
HORMAD1	1	STIL	12	CENPM	22	KIAA0586	31
HP1BP3	1	TIMP1	12	CFD	22	KIF5B	31
LMNB1	1	TOP2A	12	CROP	22	KIFC1	31
MAN1A2	1	TSC22D1	12	CTNNA1	22	LNPEP	31
MAP3K2	1	WWC1	12	DNAJC3	22	MAD2L1	31
FBXL20	1	ZWINT	12	ESCO2	22	ME3	31
KIAA0101	1	ARGLU1	13	FAM83D	22	MEGF9	31
MPHOSPH1	1	TTLL7	13	FKBP1A	22	MLF1IP	31
NIPBL	1	CEP55	14	ILF2	22	MRP63P6	31
NSUN5C	1	FAM60A	14	INADL	22	NUP43	31
NUCKS1	1	HCFC1	14	INSM1	22	OGT	31
PACS1	1	MELK	14	INTS7	22	ORMDL1	31
PIF1	1	SORL1	14	ITFG1	22	OSGIN2	31
RGS3	1	TOP2A	14	ITPR1	22	PLCXD1	31
SEPHS1	1	WDR90	14	JMJD2A	22	PSCD2	31
SH3GL2	1	ABCC2	15	KBTBD2	22	RNPC3	31
STAT5B	1	ADAMTS1	15	KIAA1712	22	RRP1	31
THRAP3	1	BIRC5	15	MAN1A2	22	SERPINB4	31
TNPO1	1	C14ORF68	15	MAP3K15	22	SKIP	31
TOMM70A	1	CAPS	15	MBD4	22	SLIT2	31
TRA2A	1	CDC27	15	MCM4	22	TSKU	31
WDR68	1	CEP57	15	MEPCE	22	ACD	32
APOA1BP	2	CHAF1B	15	MRPS2	22	ANKRD40	32
ARMC1	2	DNAJB4	15	MTMR15	22	ARHGAP8	32
ASXL1	2	FAM64A	15	NR5A2	22	BBS2	32
C12ORF32	2	FEN1	15	NUF2	22	BMP2	32
C6ORF166	2	FRZB	15	ORC1L	22	C13ORF3	32
CDC25A	2	FZR1	15	PBK	22	CCDC90B	32
CDC25C	2	GINS3	15	PLCXD1	22	CCNE1	32
CDKN1B	2	HIST2H4B	15	POLA1	22	CDC45L	32
CIITA	2	HIST2H4B	15	PTGER3	22	CKS1B	32

Gene Symbol	Cluster #	Gene Symbol	Cluster #	Gene Symbol	Cluster #	Gene Symbol	Cluster #
CIT	2	HRSP12	15	PTMS	22	DONSON	32
CKAP2	2	IL18BP	15	RAD51	22	DYNC1LI2	32
DNAJB9	2	KIAA0182	15	RBBP8	22	ERN2	32
FABP1	2	KIAA1586	15	RCCD1	22	FANCA	32
FLJ13231	2	MCM8	15	RFC4	22	FLJ20699	32
GINS2	2	NCOA3	15	RNF141	22	GOT1	32
GOLGA8A	2	NEK2	15	SETD8P1	22	HIST1H2BB	32
GSPT1	2	NUP98	15	SLBP	22	HIST3H2A	32
HERPUD2	2	PCF11	15	SMTN	22	HLA-DOA	32
HSPC157	2	POLD3	15	SP1	22	HMGB2	32
IFNAR1	2	PPP1R2	15	TXNDC9	22	HMGB3	32
IVNS1ABP	2	RANGAP1	15	UBE2D3	22	HTF9C	32
KDELC1	2	SGCD	15	ZC3HC1	22	INTS7	32
KIAA1147	2	STAT5B	15	ZNHIT2	22	ITGB3	32
KIAA1370	2	UBE2T	15	AHI1	23	KIFC2	32
KIAA1524	2	ZSCAN5	15	BARD1	23	LMBRD2	32
KIF5B	2	ATAD2	16	C15ORF23	23	MAP3K15	32
LARP7	2	CCNB2	16	INCENP	23	MDM2	32
MNX1	2	DHFR	16	LRCH1	23	MED31	32
MZF1	2	E2F5	16	BTBD3	24	NCOA5	32
NFIA	2	ENOSF1	16	C1ORF103	24	OSGIN2	32
NUDT4	2	FAM115A	16	C3ORF60	24	PDXP	32
NUSAP1	2	GADD45A	16	CCDC99	24	PHTF1	32
PANK2	2	HIF1A	16	CCNE2	24	RFC4	32
PLK2	2	HSPA8	16	CDH24	24	RNF126	32
POLQ	2	INDO	16	CENPF	24	RNF141	32
PRKAR1A	2	INSR	16	CENTB5	24	SAP30	32
PSEN1	2	KIAA0841	16	CKAP5	24	SLC38A2	32
PTPN4	2	KIAA1598	16	CTNND1	24	STCH	32
PVRIG	2	KLHDC5	16	CXCL14	24	TFF3	32
QRICH1	2	KPNB1	16	DNAJB1	24	TMEM132A	32
RAD51C	2	LBR	16	DSP	24	TPX2	32
RAN	2	MNT	16	ELP3	24	TRIP13	32
RGS3	2	NEK2	16	FREQ	24	TTK	32
RHEB	2	NEK4	16	FYN	24	TUBB2C	32
RHOBTB3	2	PMS2	16	FZR1	24	UBE2C	32
RNF113A	2	RPL13A	16	GLI1	24	UBE2C	32
SGK1	2	SDC1	16	HIST2H4B	24	BRCA1	33
TLE3	2	SFRS5	16	HS2ST1	24	C20ORF111	33
TOP1	2	SLC25A45	16	HSPB8	24	CIT	33
UACA	2	SPAG5	16	INTS8	24	DCC1	33
VPS37C	2	TFAP2A	16	KIAA1333	24	DIS3	33
WDR76	2	TGIF1	16	KIF14	24	DTL	33
PPP1R10	3	TLOC1	16	KRAS	24	DUSP4	33
AMD1	4	ZNF24	16	LMNA	24	EFHC1	33
APOA1BP	4	C9ORF100	17	MLF1IP	24	GPSM2	33

Gene Symbol	Cluster #	Gene Symbol	Cluster #	Gene Symbol	Cluster #	Gene Symbol	Cluster #
BAG3	4	CKS2	17	MSH2	24	KPNA2	33
CCDC14	4	DYNLL1	17	MUC1	24	MCM4	33
FGA	4	EIF4EBP2	17	NCOA5	24	PHF15	33
HIPK2	4	ESCO2	17	NKTR	24	ZNF281	33
KIAA1333	4	GCLM	17	OSBPL6	24	ZRANB2	33
LENG8	4	GOLGA8A	17	PCNA	24	AK3	34
NDE1	4	HIST2H4B	17	PHTF2	24	AMD1	34
NR3C1	4	HOXB4	17	PKNOX1	24	ANP32E	34
NSUN3	4	MDC1	17	POM121	24	AOC2	34
ODF2	4	MGAT2	17	PRIM1	24	ARL6IP2	34
PCNA	4	NOS1	17	PRPSAP1	24	ASAM	34
RMI1	4	NUP37	17	PRR11	24	AURKA	34
WSB1	4	PIK3CD	17	RRM2	24	CASP3	34
BRD8	5	PPP2CB	17	SCYL1	24	CCDC14	34
CDKN2D	5	PRR11	17	SMARCD1	24	CCNA2	34
DMXL2	5	RANGAP1	17	SMC4	24	CDC16	34
FANCD2	5	RNPS1	17	SUV420H1	24	CDC6	34
LYAR	5	SHCBP1	17	TIPIN	24	CKS1B	34
MATN2	5	SMARCB1	17	TOP2A	24	CLSPN	34
ABCA5	6	SNUPN	17	TUBD1	24	COQ9	34
BRD7	6	SRF	17	UBQLN2	24	CREBZF	34
C13ORF34	6	TLE3	17	UNG	24	CRK	34
C21ORF15	6	TMEM138	17	VCAM1	24	DCC1	34
CDC20	6	TNPO2	17	ANKRD10	25	DDX11	34
CDC42	6	TREX1	17	C14ORF142	25	DHX8	34
CFLAR	6	UBE2T	17	C4B	25	DNA2L	34
CIC	6	UHRF1	17	CDCA3	25	DNAJB6	34
DR1	6	WIBG	17	CRYBA1	25	DTL	34
EBI3	6	ZNF207	17	DLG7	25	E2F2	34
FAM60A	6	ABCC5	18	FUSIP1	25	EMP1	34
GATA2	6	BUB1B	18	GH1	25	EXO1	34
HELLS	6	CNOT10	18	GMNN	25	FAM110A	34
HJURP	6	DCTN6	18	GOLGA8A	25	FZR1	34
HP1BP3	6	DNAJA1	18	GTSE1	25	GOLGA8A	34
JMJD1C	6	DZIP3	18	HIST1H2AC	25	HCG_2026038	34
KIAA1524	6	FLAD1	18	KMO	25	HDAC3	34
MAPK13	6	GOLGA8A	18	PCDH7	25	HIST3H2A	34
MKI67	6	HN1	18	PPP3CA	25	HP1BP3	34
MSH2	6	KCTD2	18	PRPS2	25	KIAA1641	34
MYCBP2	6	M6PRBP1	18	PTPN9	25	KIF11	34
NLRP2	6	MCM6	18	RDH11	25	KIFC1	34
PAK1IP1	6	NEIL3	18	SRD5A1	25	LRRC17	34
PBK	6	NUP160	18	STK17B	25	LYRM7	34
PSMD11	6	OLR1	18	USP1	25	MPHOSPH1	34
TSG101	6	RRM2	18	ADAM22	26	N4BP3	34
ADCK2	7	SFRS3	18	AFAP1	26	NBPF15	34

Gene Symbol	Cluster #	Gene Symbol	Cluster #	Gene Symbol	Cluster #	Gene Symbol	Cluster #
HSPA7	7	SGK1	18	AGPAT3	26	NDC80	34
RCAN1	7	SYNCRIP	18	AMD1	26	NDE1	34
ANLN	8	TMEM138	18	ARGLU1	26	PRIM2	34
AURKB	8	TNS4	18	ARL4A	26	RANGAP1	34
C15ORF29	8	USP6NL	18	BAIAP2	26	SFPQ	34
C21ORF87	8	WDR51A	18	C14ORF57	26	SLC22A3	34
CCND1	8	WDR63	18	C9ORF100	26	SS18	34
CDC25A	8	WSB1	18	CDC25B	26	TPX2	34
CDC27	8	ANKRD25	19	CDCA7	26	TROAP	34
CIC	8	AP3D1	19	CEP70	26	UBE2S	34
DCP1A	8	ARL6IP2	19	CKAP2	26	UBL3	34
FYN	8	EFHC1	19	COQ6	26	UBXD5	34
HMMR	8	FLJ13231	19	CRLS1	26	VCL	34
ITPR3	8	IFIT1	19	G3BP1	26	WDR68	34
MASTL	8	KIAA1641	19	GCSH	26	Y18H1A.11	34
MCM2	8	MKI67	19	GRK6	26	ZMYM1	34
PRR16	8	PKMYT1	19	HIST1H2AC	26	ABCA7	35
ROCK1	8	PPP2R2A	19	HIST2H4B	26	AURKA	35
SLBP	8	PWP1	19	INSIG2	26	B4GALT1	35
SPTBN1	8	SSR3	19	KAZALD1	26	BIVM	35
ANKRD10	9	TMPO	19	KRAS	26	C4BPB	35
BUB3	9	TUBB2C	19	MCAM	26	CALD1	35
C4ORF30	9	USP53	19	MET	26	CCNF	35
C6	9	ZNF24	19	MITF	26	CDKL5	35
C9ORF140	9	ANTXR1	20	NAB1	26	CDKN2AIP	35
CALM3	9	ARHGAP19	20	PHIP	26	CENPL	35
CCND1	9	ARL6IP1	20	SAPS3	26	CHML	35
CD97	9	ASIP	20	SLC4A1AP	26	CIC	35
CDC25B	9	ATAD2	20	SMC4	26	CKS2	35
CDH24	9	BMI1	20	TLE3	26	DEPDC1	35
CHGN	9	CADM1	20	TOP3A	26	FAM113A	35
DEPDC7	9	CAPN7	20	TPX2	26	GMNN	35
DIAPH3	9	CENPQ	20	TTC31	26	HLA-DRA	35
DONSON	9	ESCO2	20	TUBA1A	26	HMGCR	35
G3BP1	9	ESD	20	TYMS	26	HTF9C	35
GDF15	9	FKBP1A	20	WDR62	26	IFIT1	35
GNB3	9	H2AFX	20	WDR68	26	KIAA0802	35
GPR126	9	HIST1H2AM	20	ZBTB7A	26	NCAPD2	35
GRPEL1	9	ITGB3BP	20	CASP2	27	NDE1	35
HDAC3	9	KIF2C	20	CBX3	27	NMB	35
HELLS	9	LMO4	20	CHAF1A	27	NUSAP1	35
HRB	9	LOC199800	20	CKS1B	27	PCF11	35
HSPA2	9	NCOA5	20	DHFR	27	PSCD3	35
HSPA8	9	RAD51AP1	20	DKC1	27	RERE	35
IDI2	9	RECQL4	20	DUSP4	27	SPAG5	35
KCTD9	9	SFRS3	20	EIF2A	27	SREBF1	35

Gene Symbol	Cluster #	Gene Symbol	Cluster #	Gene Symbol	Cluster #	Gene Symbol	Cluster #
KIF23	9	SFRS7	20	HIST2H3PS2	27	STAT5B	35
KIF5B	9	SLC25A36	20	HMG20B	27	SVIP	35
KLF6	9	SUCLG2	20	HPS4	27	TAF9	35
KLF9	9	TOB2	20	KIAA1529	27	TRIM26	35
KMO	9	TUBA3C	20	KIF22	27	TUBA4A	35
LPP	9	ZFX	20	KIF5B	27	UBE2C	35
MCM2	9	ALKBH1	21	PEBP4	27	UBE2C	35
MCM8	9	AP3M2	21	PNN	27	VANGL1	35
MCM5	9	ASF1B	21	POLD3	27	VEGFC	35
NFE2L2	9	ASPHD2	21	RAB23	27	ZCCHC10	35
NFIC	9	ATF7IP	21	RBM8A	27	ZNF521	35
NUF2	9	BRIP1	21	SLC38A2	27	CCRK	36
PASK	9	C15ORF29	21	SV2B	27	DMTF1	36
PCAF	9	CDC2	21	AP4B1	28	RPS25	36
PLK1	9	CDK7	21	ASXL1	28	UHRF1	37
PSMG3	9	CTCF	21	BRD8	28	AKAP13	38
PTTG1	9	CYB5R2	21	C14ORF106	28	ANP32B	38
RRM1	9	DEPDC1B	21	CCNB1	28	ANP32E	38
RUNX1	9	DHFR	21	CDK7	28	C16ORF57	38
SCML1	9	E2F1	21	CEP350	28	C1ORF2	38
SHC1	9	EIF4E	21	CNTROB	28	CASP2	38
SLC25A27	9	ESPL1	21	DEXI	28	CDKN2AIP	38
TOPBP1	9	FAM105B	21	KIAA1333	28	CDR2	38
TTC31	9	FXR1	21	RSRC2	28	CHEK2	38
USP1	9	GAS1	21	TRAIP	28	CTR9	38
USP16	9	GAS6	21	ZPBP	28	DNAJB1	38
VANGL1	9	GATA2	21	CCNA2	29	ECT2	38
WSB1	9	GNB1	21	CDC42EP1	29	FADD	38
YWHAH	9	HIST2H4B	21	CDKN2C	29	FEM1B	38
ZNF593	9	HMG20B	21	DLG7	29	FRS2	38
ACYP1	10	HSPB8	21	GABPB2	29	GPR126	38
CD24	10	JARID1B	21	HELLS	29	GTSE1	38
CDC42EP4	10	MAP3K7IP2	21	KATNA1	29	HMGB2	38
CREBZF	10	MCM6	21	KIAA1586	29	HSF2	38
HIST2H2AA3	10	MDM1	21	MAP3K15	29	IFIT1	38
KIAA0182	10	NASP	21	MCM6	29	KCNC4	38
KPNA2	10	NFKBIL2	21	MND1	29	MLLT4	38
TAF15	10	NUCKS1	21	MZF1	29	NY-SAR-48	38
TSN	10	ODF2	21	NPAT	29	ORC3L	38
WSB1	10	OGT	21	PPP1R10	29	PLAG1	38
ANLN	11	PTP4A1	21	PRC1	29	RAD18	38
HJURP	11	RAB3A	21	RPA2	29	RFC2	38
ACPP	12	RAD54L	21	STAG1	29	RPS25	38
ASXL1	12	REEP1	21	TCERG1	29	SLC39A10	38
ATF7IP	12	SAP30BP	21	TMEM140	29	SLC44A2	38
AURKB	12	SGK1	21	VANGL1	29	SLC9A3	38

Gene Symbol	Cluster #	Gene Symbol	Cluster #	Gene Symbol	Cluster #	Gene Symbol	Cluster #
BCLAF1	12	SP1	21	ANP32E	30	STAG3L2	38
C6ORF166	12	TRIM45	21	CFLAR	30	TACC3	38
CASP8AP2	12	TUBB2A	21	E2F1	30	TOMM34	38
CLSPN	12	USP13	21	KIF23	30	TULP4	38
DET1	12	VANGL1	21	MCM4	30	TXNRD1	38
FANCG	12	VPS25	21	MCM5	30	USP1	38
GOLGA8A	12	XPO4	21	WISP1	30	ZNF217	38
HN1	12	ZBED5	21	ADH4	31	ZNF587	38
