# Supplementary material for: Reconstruction of Gene Regulatory Modules in Cancer Cell Cycle by Multi-Source Data Integration
Source: PLoS One. 2010 Apr 21;5(4):e10268. doi: 10.1371/journal.pone.0010268 (PMC2858157; doi:10.1371/journal.pone.0010268)
Supplement: Table S3 — A list of 46 transcription factors in human cell cycle selected as candidates to regulate downstream target genes. (0.03 MB PDF) [file pone.0010268.s003.pdf]

Table S3 – List of 46 transcription factors in human cell cycle selected as candidates to regulate downstream target genes

Gene Name	IMAGE ID	GO number	Associated GO category	Specific role during transcription
BCLAF1	**173309**	GO:0045449	Regulation of transcription	Transcriptional repressor
BRCA1	**241474**	GO:0045449	Regulation of transcription	Transcriptional activator/repressor
BRD8	**815287**	GO:0003700	Transcription factor activity	Transcriptional coactivator
CDK7	**1915416**/130242	GO:0045449	Regulation of transcription	Transcriptional activator
CIITA	**1536451**	GO:0045449	Regulation of transcription	Transcriptional activator/repressor
CTCF	**240367**	GO:0003700	Transcription factor activity	Transcriptional activator/repressor
DMTF1	**490728**	GO:0003700	Transcription factor activity	Transcriptional activator
DR1	**487797**/566760	GO:0045449	Regulation of transcription	Transcriptional repressor
DSCR1	**884462**	GO:0003700	Transcription factor activity	Transcriptional repressor
E2F1	**236142**/768260	GO:0003700	Transcription factor activity	Transcriptional activator
E2F2	**293331**	GO:0003700	Transcription factor activity	Transcriptional activator
FOXM1	**564803**	GO:0003700	Transcription factor activity	Transcriptional activator
GATA2	**149809**/135688	GO:0003700	Transcription factor activity	Transcriptional activator
GTF3C4	**780958**/291827	GO:0005667	Transcription factor complex	Transcriptional activator
HCFC1	**344049**	GO:0003700	Transcription factor activity	Transcriptional activator/repressor
HIF1A	**897806**	GO:0003700	Transcription factor activity	Transcriptional activator
HMG20B	**878184**	GO:0003700	Transcription factor activity	Transcriptional repressor
HMGB2	**1842250**/363103	GO:0003700	Transcription factor activity	Transcriptional repressor
ILF2	**242952**	GO:0045449	Regulation of transcription	Transcriptional activator
KDM5B	**838829**	GO:0003700	Transcription factor activity	Transcriptional repressor
KLF6	**510381**	GO:0003700	Transcription factor activity	Transcriptional activator
KLF9	**302549**	GO:0003700	Transcription factor activity	Transcriptional activator/repressor
MAPK13	**590774**	GO:0045449	Regulation of transcription	Transcriptional repressor
MNT	**809731**	GO:0003700	Transcription factor activity	Transcriptional repressor
NCOA3	**197520**	GO:0045449	Regulation of transcription	Transcriptional coactivator
NFE2L2	**884438**	GO:0003700	Transcription factor activity	Transcriptional activator
NFIC	**1455463**/265874	GO:0003700	Transcription factor activity	Transcriptional activator
NR3C1	**271198**	GO:0003700	Transcription factor activity	Transcriptional coactivator
NR5A2	**245517**	GO:0003700	Transcription factor activity	Transcriptional activator
PCNA	**43229**/789182	GO:0006275	Regulation of transcription	Transcriptional repressor
PHTF2	**30114**	GO:0045449	Regulation of transcription	Transcriptional activator/repressor
PKNOX1	**1947972**	GO:0003700	Transcription factor activity	Transcriptional activator
PTTG1	**2018976**/781089	GO:0003700	Transcription factor activity	Transcriptional activator/repressor
RBPJ	**845502**	GO:0045449	Regulation of transcription	Transcriptional repressor

Gene Name	IMAGE ID	GO number	Associated GO category	Specific role during transcription
SCML1	**202704**	GO:0003700	Transcription factor activity	Transcriptional repressor
SP1	**782622/**80318	GO:0003700	Transcription factor activity	Transcriptional activator
SRF	**840636**	GO:0003700	Transcription factor activity	Transcriptional activator
STAT1	**545503**	GO:0003700	Transcription factor activity	Transcriptional activator
STAT5B	**134120/**712840/ 132857	GO:0003700	Transcription factor activity	Transcriptional activator
TCERG1	**272192**	GO:0045449	Regulation of transcription	Transcriptional coactivator
TFAP2A	**137387**	GO:0003700	Transcription factor activity	Transcriptional activator/repressor
TSC22D1	**868630**	GO:0003700	Transcription factor activity	Transcriptional repressor
UHRF1	**366414/**1550739	GO:0003700	Transcription factor activity	Transcriptional activator
ZNF207	**246869**	GO:0003700	Transcription factor activity	Transcriptional activator/repressor
ZNF24	**296429**	GO:0003700	Transcription factor activity	Transcriptional repressor
ZNF281	**280750**	GO:0003700	Transcription factor activity	Transcriptional repressor

The table lists the transcription factors through their gene names and IMAGE IDs that refer to the probes used in the original microarray data (Whitfield et al. 2002). The expression profiles of bolded IMAGE IDs were used in further prediction steps.

References

Whitfield ML, Sherlock G, Saldanha AJ, Murray JI, Ball CA et al. (2002) Identification of genes periodically expressed in the human cell cycle and their expression in tumors. Mol Biol Cell 13(6): 1977-2000.
