# Supplementary material for: Reconstruction of Gene Regulatory Modules in Cancer Cell Cycle by Multi-Source Data Integration
Source: PLoS One. 2010 Apr 21;5(4):e10268. doi: 10.1371/journal.pone.0010268 (PMC2858157; doi:10.1371/journal.pone.0010268)
Supplement: Table S4 — Identified E2F target genes. (0.04 MB PDF) [file pone.0010268.s004.pdf]

E2F Target Genes Listed in Bracken et al. (2004) and Present in the List of Genes Analyzed	Included in the Gene Modules Identfied (Y/N)
AURKB	N
BARD1	N
BMP2	N
BRCA1	N
BUB1	N
BUB1B	N
BUB3	N
CASP3	N
CCNA2	Y
CCND1	Y
CCNE1	Y
CCNE2	N
CDC2	Y
CDC20	N
CDC25A	Y
CDC45L	Y
CDC6	Y
CDKN2C	Y
CDKN2D	N
CENPE	N
CKS2	N
DHFR	N
E2F1	Y
E2F2	Y
FEN1	N
MAD2L1	N
MCM2	Y
MCM4	Y
MCM5	Y
MCM6	Y
MSH2	N
NPAT	Y
ORC1L	N
PCNA	N
PMS2	N
PRC1	N
RAD51	N
RAD54L	N
RFC2	N
RFC4	N
RPA2	N
RRM1	N
RRM2	N
TOP2A	N
TTK	N
TYMS	N
