# Supplementary figures and images for: Reconstruction of Gene Regulatory Modules in Cancer Cell Cycle by Multi-Source Data Integration
Source: PLoS One. 2010 Apr 21;5(4):e10268. doi: 10.1371/journal.pone.0010268 (PMC2858157; doi:10.1371/journal.pone.0010268)

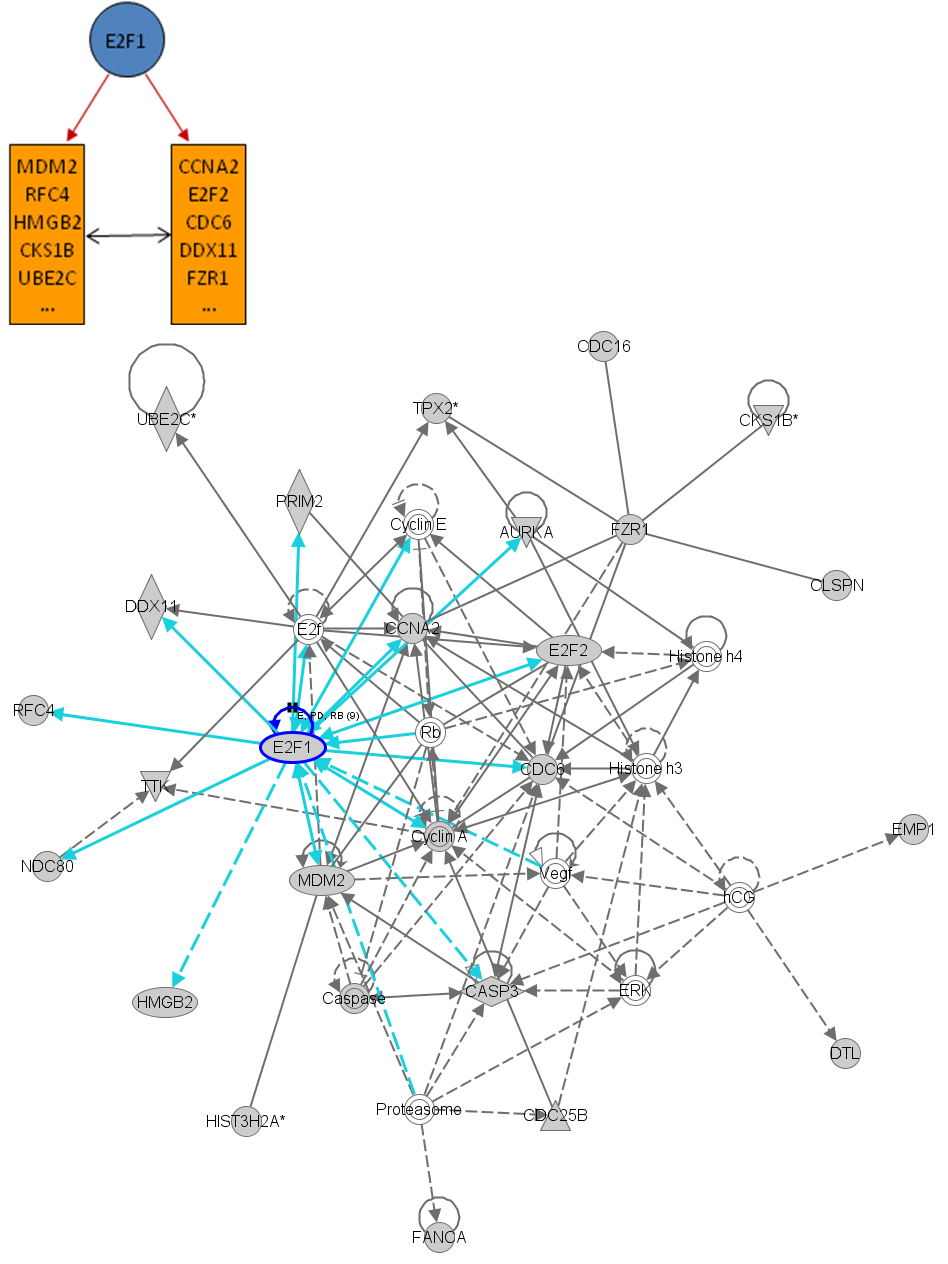

Supplement: Figure S1 — Ingenuity analysis result for a predicted network motif. (0.24 MB TIF) [file pone.0010268.s005.tif]

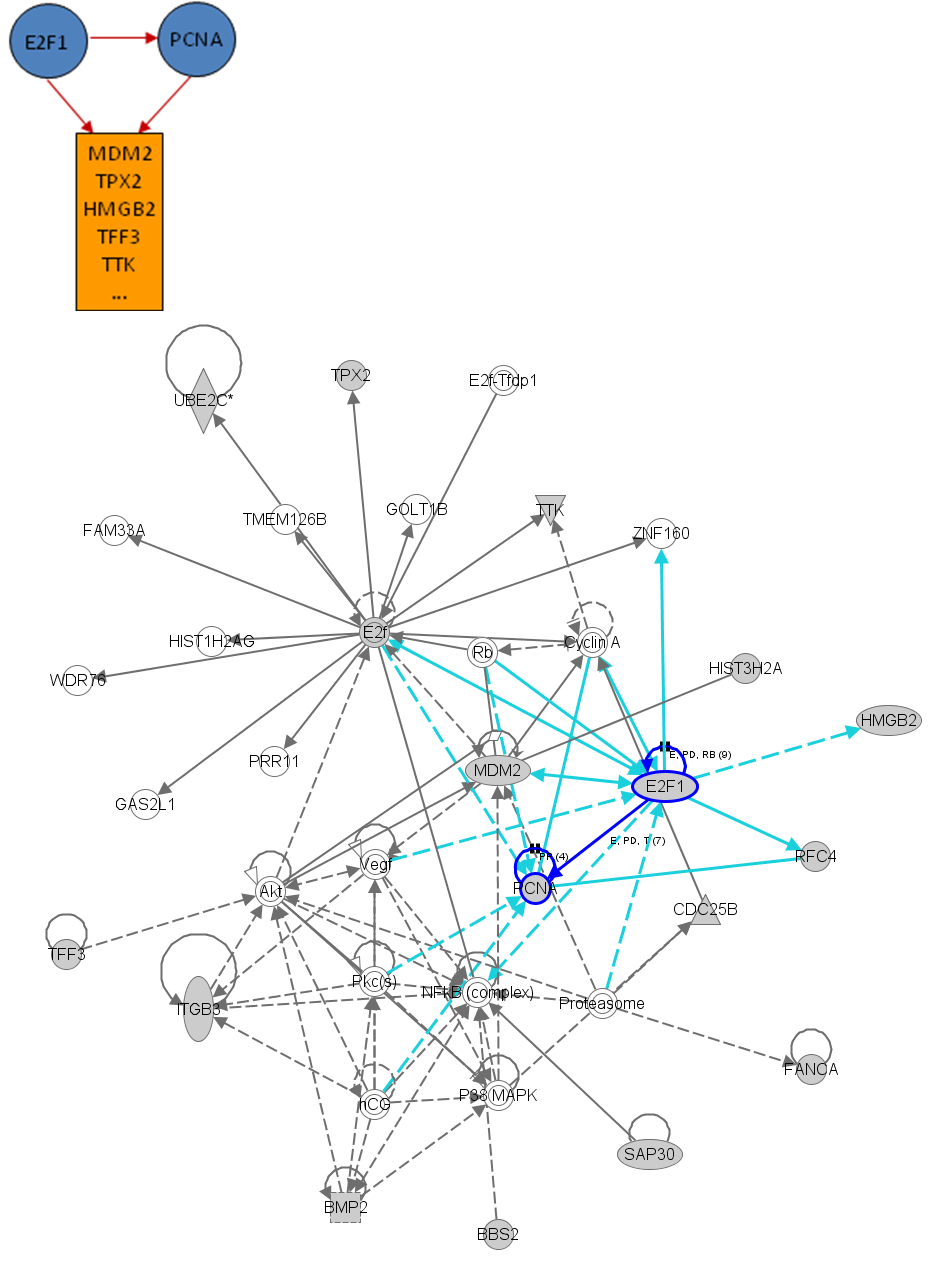

Supplement: Figure S2 — Ingenuity analysis result for a predicted network motif. (0.22 MB TIF) [file pone.0010268.s006.tif]

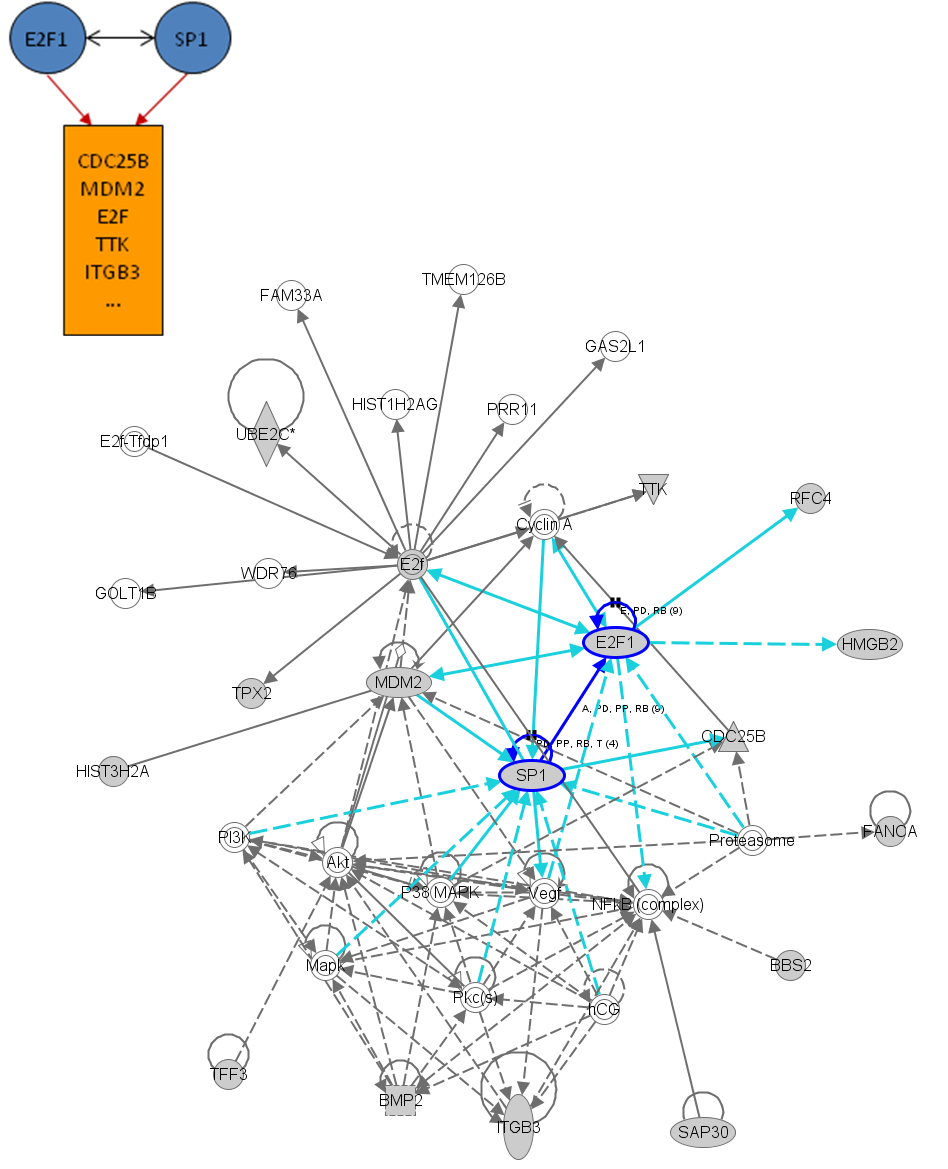

Supplement: Figure S3 — Ingenuity analysis result for a predicted network motif. (0.23 MB TIF) [file pone.0010268.s007.tif]

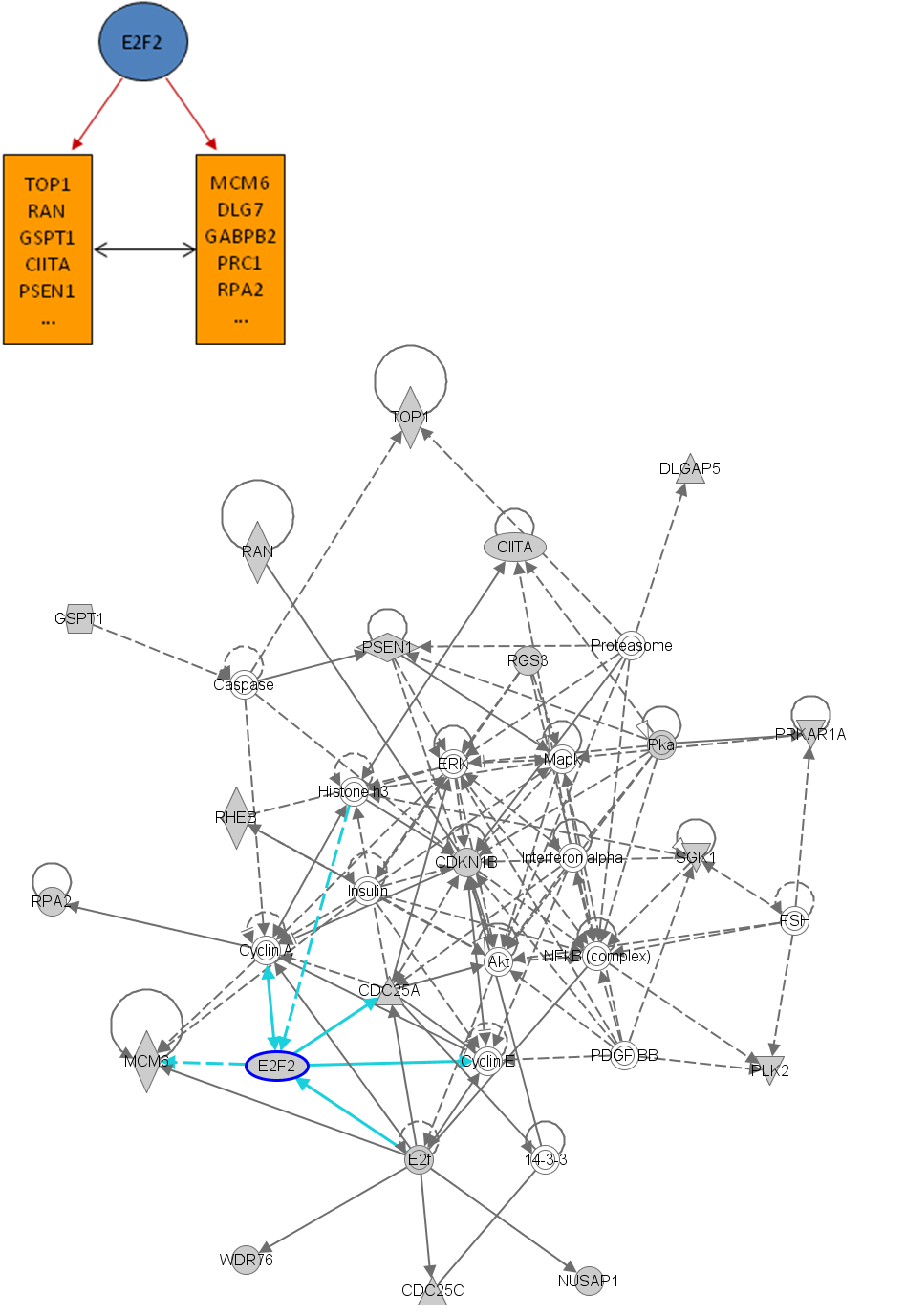

Supplement: Figure S4 — Ingenuity analysis result for a predicted network motif. (0.25 MB TIF) [file pone.0010268.s008.tif]

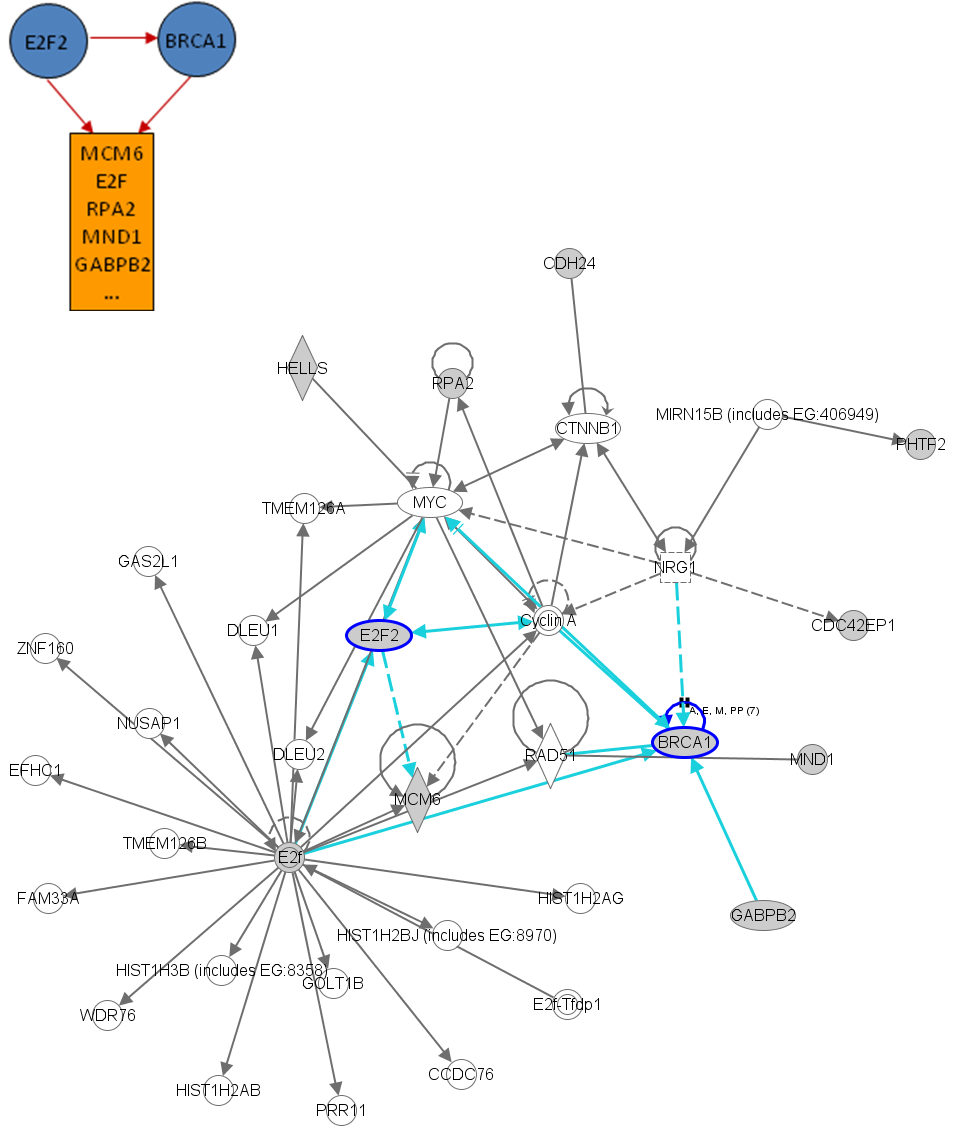

Supplement: Figure S5 — Ingenuity analysis result for a predicted network motif. (0.19 MB TIF) [file pone.0010268.s009.tif]

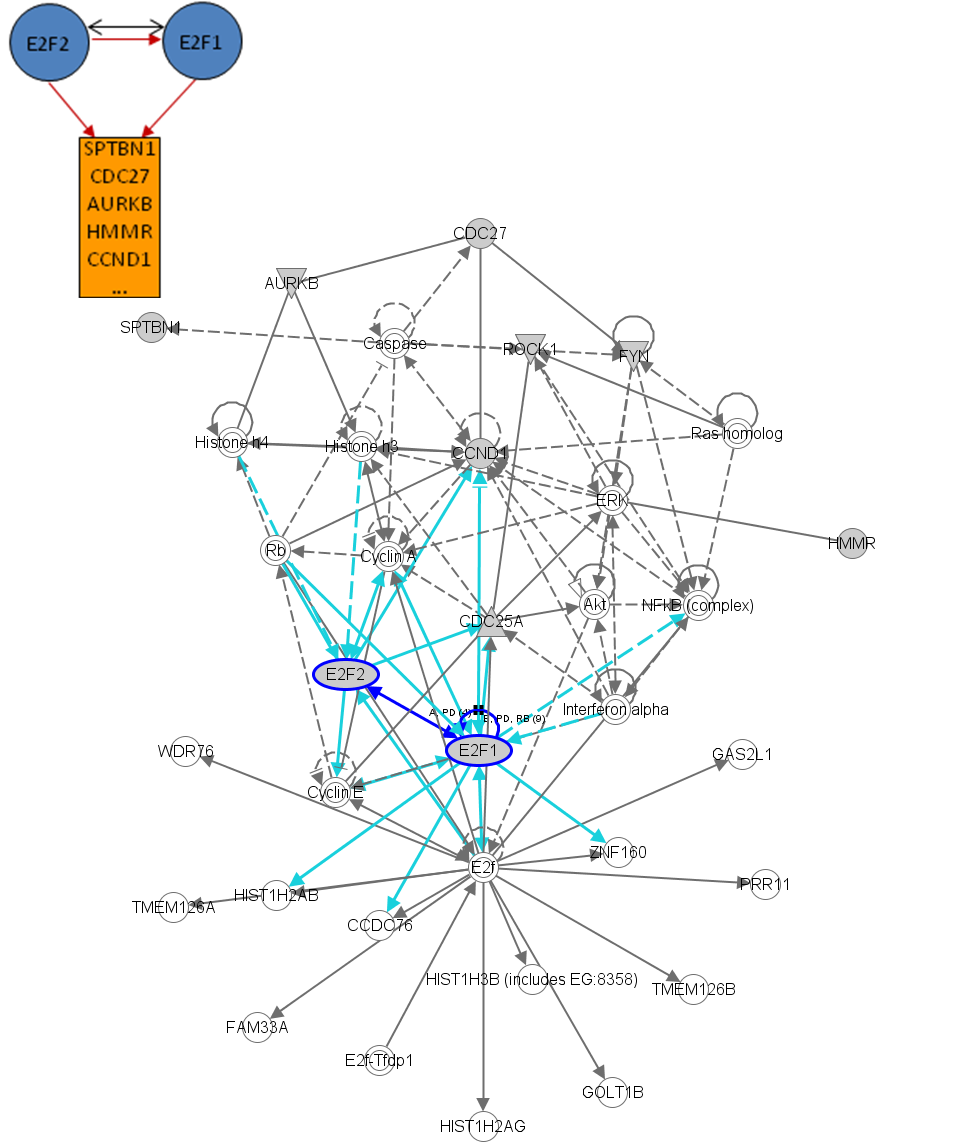

Supplement: Figure S6 — Ingenuity analysis result for a predicted network motif. (0.22 MB TIF) [file pone.0010268.s010.tif]

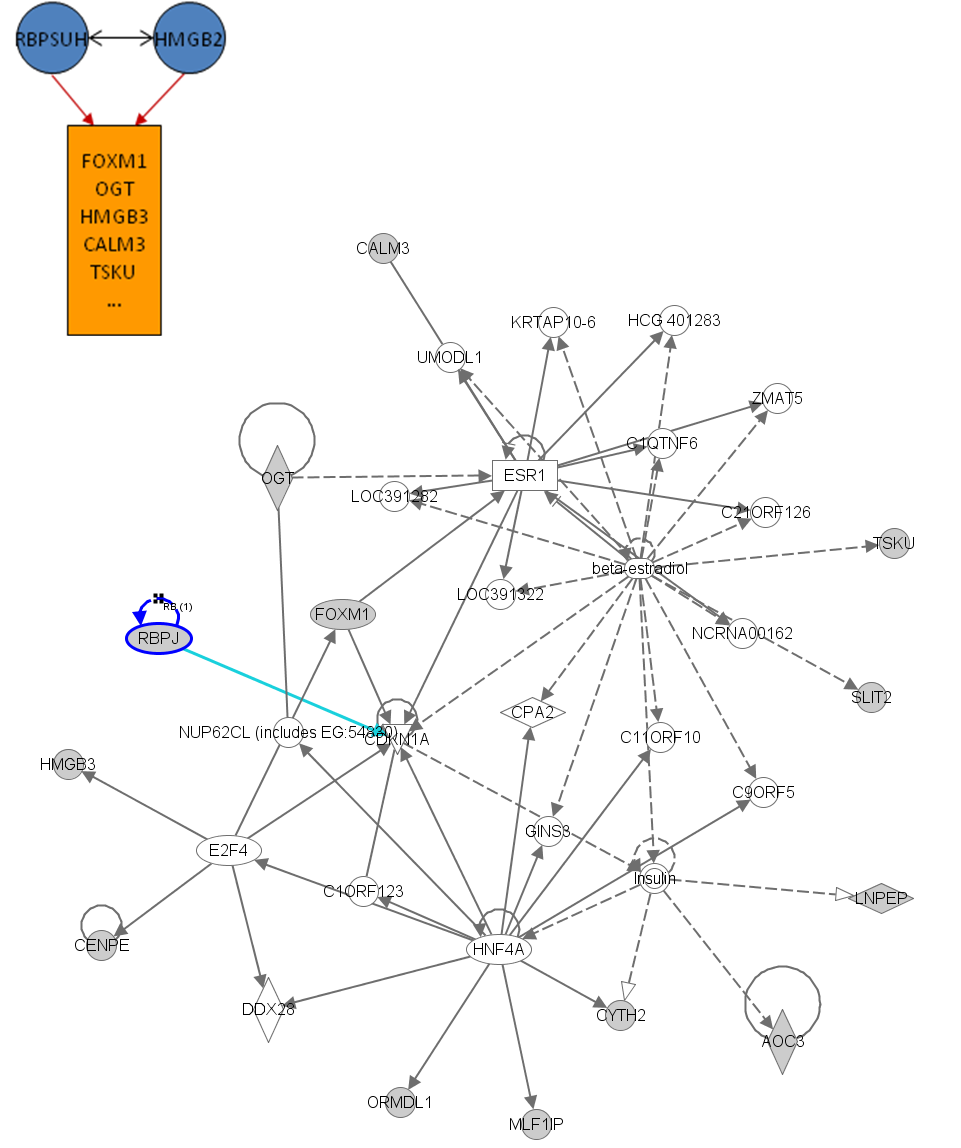

Supplement: Figure S7 — Ingenuity analysis result for a predicted network motif. (0.19 MB TIF) [file pone.0010268.s011.tif]

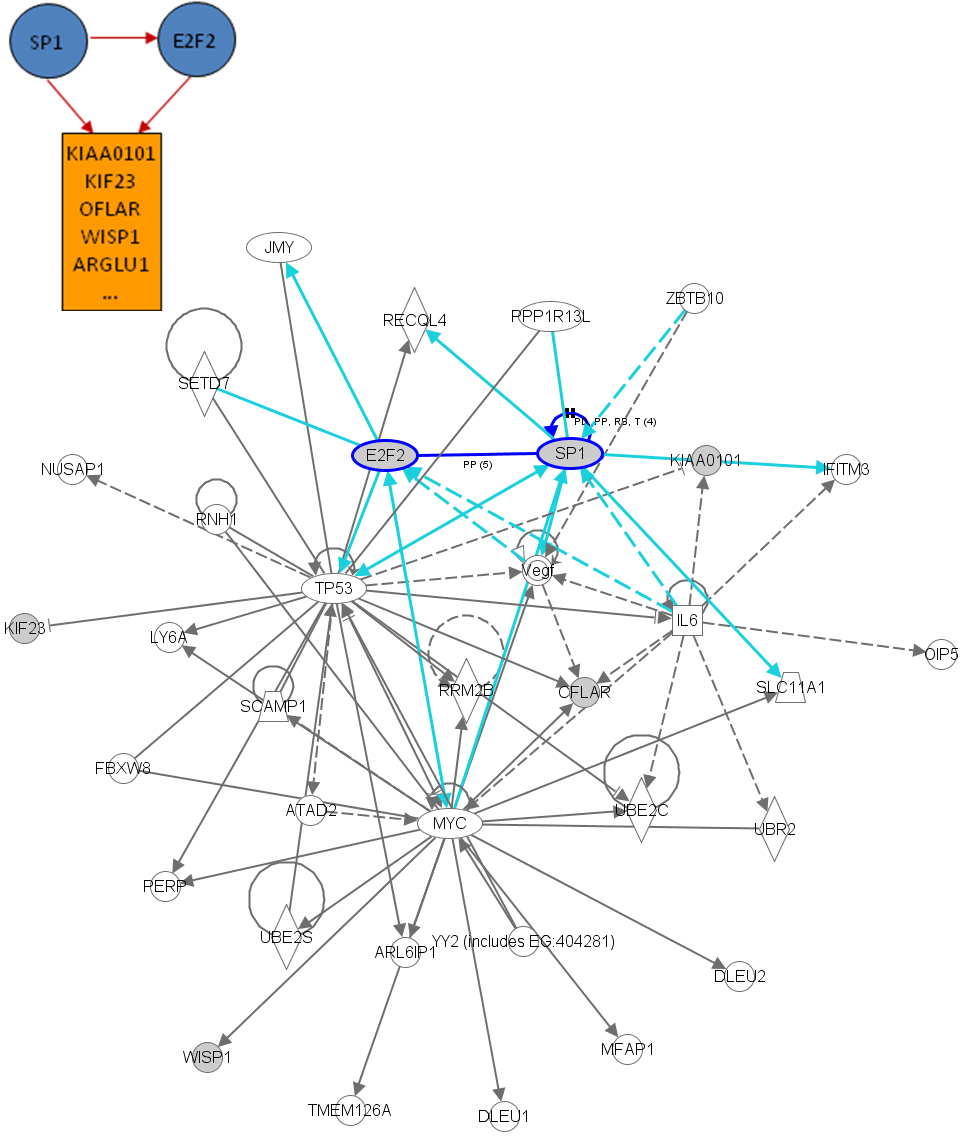

Supplement: Figure S8 — Ingenuity analysis result for a predicted network motif. (0.22 MB TIF) [file pone.0010268.s012.tif]
